# Biomimetic Remodeling of Microglial Riboflavin Metabolism Ameliorates Cognitive Impairment by Modulating Neuroinflammation

**DOI:** 10.1002/advs.202300180

**Published:** 2023-02-17

**Authors:** Mengran Zhang, Huaqing Chen, Wenlong Zhang, Yan Liu, Liuyan Ding, Junwei Gong, Runfang Ma, Shaohui Zheng, Yunlong Zhang

**Affiliations:** ^1^ Department of Neurology Institute of Neuroscience Key Laboratory of Neurogenetics and Channelopathies of Guangdong Province and the Ministry of Education of China The Second Affiliated Hospital Guangzhou Medical University Guangzhou 510260 China; ^2^ Shenzhen Key Laboratory of Gene and Antibody Therapy Center for Biotechnology and Biomedicine State Key Laboratory of Chemical Oncogenomics State Key Laboratory of Health Sciences and Technology Institute of Biopharmaceutical and Health Engineering Shenzhen International Graduate School Tsinghua University Shenzhen Guangdong 518055 China; ^3^ Department of Neurology The First Affiliated Hospital of Guangzhou Medical University Guangzhou 510120 China; ^4^ School of Traditional Chinese Medicine Jinan University Guangzhou 510632 China

**Keywords:** cognitive decline, flavin mononucleotide, microglial targeted delivery, Neuroinflammation, riboflavin kinase

## Abstract

Neuroinflammation, for which microglia are the predominant contributors, is a significant risk factor for cognitive dysfunction. Riboflavin (also known as vitamin B2) ameliorates cognitive impairment via anti‐oxidative stress and anti‐inflammation properties; however, the underlying mechanisms linking riboflavin metabolism and microglial function in cognitive impairment remain unclear. Here, it is demonstrated that riboflavin kinase (RFK), a critical enzyme in riboflavin metabolism, is specifically expressed in microglia. An intermediate product of riboflavin, flavin mononucleotide (FMN), inhibited RFK expression via regulation of lysine‐specific methyltransferase 2B (KMT2B). FMN supplementation attenuated the pro‐inflammatory TNFR1/NF‐*κ*B signaling pathway, and this effect is abolished by KMT2B overexpression. To improve the limited anti‐inflammatory efficiency of free FMN, a biomimetic microglial nanoparticle strategy (designated as MNPs@FMN) is established, which penetrated the blood brain barrier with enhanced microglial‐targeted delivery efficiency. Notably, MNPs@FMN ameliorated cognitive impairment and dysfunctional synaptic plasticity in a lipopolysaccharide‐induced inflammatory mouse model and in a 5xFAD mouse model of Alzheimer's disease. Taken together, biomimetic microglial delivery of FMN may serve as a potential therapeutic approach for inflammation‐dependent cognitive decline.

## Graphical abstract

A biomimetic microglial nanoparticle system (MNPs@FMN) is established for precise microglial delivery of flavin mononucleotide (FMN) to ameliorate cognitive impairment and restore synaptic plasticity in inflammation‐based cognitive decline mouse models. Mechanistically, MNPs@FMN alleviated pro‐inflammatory response by inhibiting the TNFR1/NF‐*κ*B signaling pathway, for which this neuroprotection may be involved in the regulation of microglial expressed riboflavin kinase (RFK) via KMT2B.

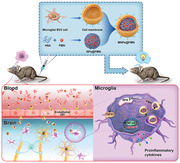

## 1 Introduction

As resident immune cells in the central nervous system (CNS), microglia play key roles in modulating cognitive function. Microglia stimulate learning‐related synapse formation in the healthy adult brain and maintain neuronal connectivity and synaptic homeostasis for learning and memory via synaptic pruning.^[^ ^1^ ^]^ They also preserve cognitive function in neurodegenerative diseases by engulfing cellular debris and misfolded proteins, such as amyloid *β* (A*β*), tau and *α*‐synuclein.^[^ ^2^ ^]^ Additionally, microglia are major players in neuroinflammation, with their overactivation substantially increasing the production of cytokines and reactive oxygen species (ROS).^[^ ^3^, ^4^ ^]^ Neuroinflammation drives the progression of cognitive impairment‐related diseases, including neurodegenerative disorders such as Alzheimer's disease (AD) and Lewy body dementia^[^ ^3^, ^5^ ^]^; and neurological disorders such as traumatic brain injury or stroke‐associated cognitive dysfunction.^[^ ^6^ ^]^ Thus, the regulation of microglia provides a promising approach to improve inflammation‐related cognitive dysfunction.

Metabolism shapes microglial function in stimulating learning and memory as well as inflammation‐related cognitive decline.^[^ ^7^ ^]^ Notably, riboflavin metabolism plays a vital role in regulating cognitive function; for example, low levels of vitamin B have been observed in patients with dementia and AD, and supplementation of vitamin B has proven beneficial in treating patients with cognitive impairment.^[^ ^8^ ^]^ Among the beneficial effects, riboflavin (vitamin B2) protects cells from oxidative stress by enhancing antioxidant enzyme activities and the glutathione redox cycle and reducing pro‐inflammatory responses in the brain.^[^ ^9^ ^]^ Low levels of vitamin B expression in the brains of AD patients may be attributed in part to low levels of a key enzyme in riboflavin metabolism, riboflavin kinase (RFK), and supplementation of a metabolic product of RFK, flavin mononucleotide (FMN), suppresses A*β* toxicity by regulating redox status.^[^ ^10^ ^]^ RFK also mediates the activity of tumor necrosis factor (TNF)‐activating nicotinamide adenine dinucleotide phosphate (NADPH) oxidase via coupling with TNF receptor‐1 (TNFR1).^[^ ^11^ ^]^ Thus, we hypothesized that RFK may contribute to the inflammatory state associated with cognitive impairment.

**Scheme 1:**
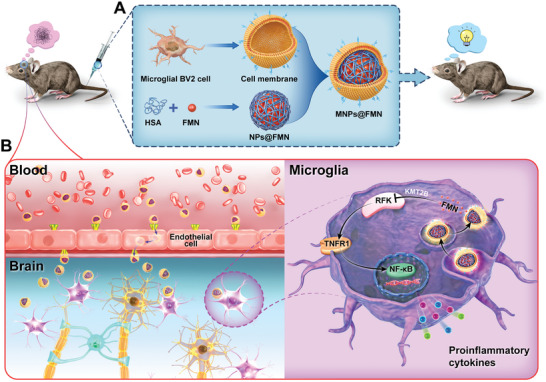
Schematic illustration showing the preparation and underlying mechanism of MNPs@FMN in memory protection. A) Preparation process of biomimetic nanocarriers (MNPs@FMN). B) Schematic diagram showing the proposed mechanism of MNPs@FMN crossing the BBB to become recruited by microglia. Briefly, MNPs@FMN penetrates the BBB via binding cell surface receptors on the brain endothelial cells. Afterward, MNPs@FMN accumulates in microglia, and released FMN inhibits RFK via KMT2B, while RFK promotes the TNFR1/NF‐*κ*B signaling pathway. Ultimately, MNPs@FMN restores cognitive function by suppressing the inflammatory response.

The objective of this study was to examine the role of microglial riboflavin metabolism in cognitive dysfunction. Here, we report that RFK is specifically expressed in microglia, and that its expression is enhanced by pro‐inflammatory events. As an intermediate product of riboflavin metabolism, FMN inhibits RFK expression, while the ultimate downstream metabolite, flavin adenine dinucleotide (FAD), has no effect. Genetic knockdown of *Rfk* or supplementation of FMN attenuates the pro‐inflammatory TNFR1/NF‐*κ*B signaling pathway. Moreover, the effects of FMN on RFK expression rely on the regulation of lysine‐specific methyltransferase 2B (KMT2B). Because intraperitoneal injection of FMN showed limited anti‐inflammatory effects in a lipopolysaccharide (LPS)‐induced mouse model, we establish a biomimetic microglial nanoparticle (BMNP) platform to specifically deliver FMN to the microglia. The BMNP strategy (termed MNPs@FMN) was designed with an encapsulated FMN‐coated human serum albumin (HSA) nanoparticles core and a microglial BV2 cell membrane shell (**Scheme** **1**A). Our results demonstrate that MNPs@FMN can successfully penetrate the blood‐brain barrier (BBB) and facilitate microglial targeted delivery (Scheme 1B). Importantly, MNPs@FMN ameliorated cognitive dysfunction, impaired synaptic plasticity, and inflammatory response in the LPS‐induced mouse model and in a 5xFAD mouse model of AD. Taken together, our results suggest that microglial supplementation of FMN may serve as a novel therapeutic intervention for inflammation‐based cognitive decline.

## 2 Results

### 2.1 Microglial RFK Contributes to the LPS‐Induced Inflammatory Response

To investigate inflammation‐based cognitive impairment, we first established an LPS‐induced mouse model. Behavioral performances of LPS‐treated mice in the open field test (OFT), Y maze and Morris water maze suggest that the exploratory ability, working memory, and spatial memory were impaired (Figure S1A‐N, Supporting Information). Furthermore, LPS promoted the mRNA expression of *Cx3cr1* (fold‐change [FC] = 1.240, *p* = 0.0006), *Csf1r* (FC = 1.389, *p* = 0.0005), *P2ry12* (FC = 1.197, *p* = 0.0030), *Il‐1b* (FC = 1.732, *p* = 0.0116), *Il‐6* (FC = 1.427, *p* = 0.0131) and *Tnfa* (FC = 1.415, *p* = 0.0006) in the hippocampus (Figure S2A and B, Supporting Information), and it also increased the hippocampal protein expression of IL‐1*β* (FC = 1.369, *p* = 0.0021), IL‐6 (FC = 1.386, *p* = 0.0024) and TNF‐*α* (FC = 1.382, *p* = 0.0026) (Figure S2C‐E, Supporting Information). Consistently, the volume of ionized calcium binding adapter molecule (Iba1)‐positive cells was increased (FC = 1.527, *p* < 0.0001), while the process complexity and endpoint voxels were reduced (process complexity: FC = 0.463, *p* < 0.0001; endpoint voxels: FC = 0.596, *p* = 0.0004) (Figure S2F,G, Supporting Information), which suggests that LPS promotes a change in the microglia from a “resting” phenotype to an “activated” phenotype.

**Figure 1:**
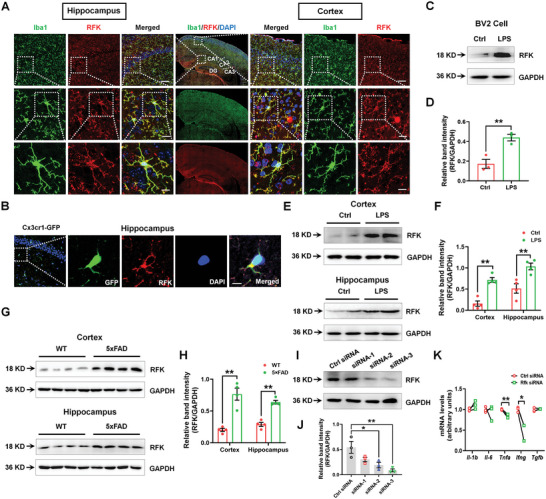
RFK is mainly located in microglia and its expression is increased in the inflammation‐based cognitive impairment model. A) Immunofluorescence staining of RFK with Iba1 in the CA1 of the hippocampus and cortex. Scale bars, 50 µm (top), 10 µm (middle), and 5 µm (bottom). B) Immunofluorescence staining of RFK with GFP in the hippocampi of Cx3cr1‐GFP mice. Scale bars, 8 µm. C and D) Representative blots and quantification showing RFK expression levels in Control and LPS‐treated BV2 cells *n* = 3 per group. E,F) Representative blots and quantification showing RFK expression levels in the cortex and hippocampus of Control mice and the LPS mouse model *n* = 4 per group. G,H) Representative blots and quantification showing RFK expression levels in the cortex and hippocampus of WT mice and the 5xFAD mouse model *n* = 4 per group. I,J) Representative blots and quantification showing RFK expression levels in BV2 cells treated with Control siRNA and three targeting RFK siRNAs *n* = 3 per group. (K) Effects of Rfk siRNA on the mRNA expression levels of *Il‐1b*, *Il‐6*, *Tnfa*, *Ifng*, and *Tgfb* in BV2 cells *n* = 3 per group. Results are expressed as mean ± SEM. ^**^ *p* < 0.01, *p < 0.05 versus Ctrl or Ctrl siRNA or WT. Statistical significance was determined using Student's *t*‐test.

To address how riboflavin metabolism acts on microglial function in cognitive decline, we examined the expression pattern of RFK in neural cells. RFK expression was detected in Iba1‐positive cells, but not NeuN or glial fibrillary acidic protein (GFAP)‐positive cells in the hippocampus and cortex (**Figure** **1**A, Figures S3 and S4, Supporting Information), suggesting that RFK in the brain is mainly expressed in the microglia. As additional verification, RFK co‐localized with GFP expressed under the control of a Cx3cr1 (classic microglial marker) promoter in Cx3cr1‐GFP reporter genetic mice (Figure 1B). Furthermore, RFK expression was increased in LPS‐treated microglial BV2 cells (FC = 2.540, *p* = 0.0093) and in the cortex and hippocampus of LPS‐induced mice (cortex: FC = 4.550, *p* = 0.0006; hippocampus: FC = 2.016, *p* = 0.0086) and 5xFAD mice (cortex: FC = 3.787, *p* = 0.0012; hippocampus: FC = 2.210, *p* = 0.0002) (Figure 1C–H). To evaluate the function of RFK in the LPS‐induced inflammatory response, we designed small interference RNAs (siRNAs) to suppress RFK expression (Figure 1I,J). Our results demonstrate that *Rfk* knockdown with siRNA‐3 (hereafter referred to as “Rfk siRNA”) decreased the gene expression of *Tnfa* and *Ifng* (*Tnfa*: FC = 0.844, *p* = 0.0035; *Ifng*: FC = 0.488, *p* = 0.0166) (Figure 1K), further indicating that RFK may play a role in TNF‐*α*‐associated inflammation.

**Figure 2:**
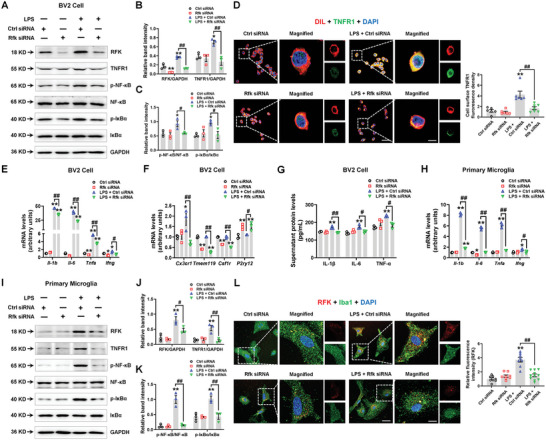
Microglial RFK contributes to LPS‐induced inflammation via the TNFR1/NF‐*κ*B pathway. A‐C) Representative blots and quantification showing RFK, TNFR1, p‐NF‐*κ*B, NF‐*κ*B, p‐I*κ*B*α* and I*κ*B*α* expression levels in LPS‐treated BV2 cells with Control siRNA or Rfk siRNA *n* = 3 per group. D) Immunofluorescence staining and quantification of TNFR1 with the membrane dye DIL in LPS‐treated BV2 cells with Control siRNA or Rfk siRNA *n* = 6–7. Scale bars, 20 µm. Magnified images are shown in the right columns. Scale bars, 5 µm. E,F) The mRNA expression levels of *Il‐1b*, *Il‐6*, *Tnfa*, *Ifng*, *Cx3cr1*, *Tmem119*, *Csf1r*, and *P2ry12* in LPS‐treated BV2 cells with Control siRNA or Rfk siRNA *n* = 3 per group. G) The secretion of IL‐1*β*, IL‐6, and TNF‐*α* in the supernatants of LPS‐treated BV2 cells with Control siRNA or Rfk siRNA *n* = 3 per group. H) The mRNA expression levels of *Il‐1b*, *Il‐6*, *Tnfa*, and *Ifng* in LPS‐treated primary microglia with Control siRNA or Rfk siRNA *n* = 3 per group. I–K) Representative blots and quantification showing RFK, TNFR1, p‐NF‐*κ*B, NF‐*κ*B, p‐I*κ*B*α* and I*κ*B*α* expression levels in LPS‐treated primary microglia with Control siRNA or Rfk siRNA *n* = 3 per group. L) Immunofluorescence staining and quantification of RFK with Iba1 in LPS‐treated primary microglia with Control siRNA or Rfk siRNA *n* = 8–11. Scale bars, 40 µm. Magnified images are shown in the right columns. Scale bars, 13 µm. Results are expressed as mean ± SEM. ^**^ *p* < 0.01, *p < 0.05 versus Ctrl siRNA; ^##^ *p* < 0.01, ^#^ *p* < 0.05 versus LPS + Ctrl siRNA. Statistical significance was determined using One‐way ANOVA followed by Tukey's *post‐hoc* test.

RFK has previously been reported to couple TNFR1 to active NADPH oxidase.^[^ ^11^ ^]^ Therefore, we assessed the role of RFK in the LPS‐induced TNFR1/NF‐*κ*B signaling pathway. *Rfk* knockdown decreased RFK and TNFR1 expression (RFK: FC = 0.326, *p* < 0.0001; TNFR1: FC = 0.380, *p* = 0.0040), as well as the phosphorylation of NF‐*κ*B and I*κ*B*α* in LPS‐treated microglial BV2 cells (p‐NF‐*κ*B: FC = 0.639, *p* = 0.0478; p‐I*κ*B*α*: FC = 0.544, *p* = 0.0452) (**Figure** **2**A–C). We also verified our results by staining TNFR1 with cellular membrane probes (DIL) (Figure 2D). Because TNFR1/NF‐*κ*B is associated with LPS‐induced inflammation and NF‐*κ*B is responsible for the transcription of pro‐inflammatory cytokines,^[^ ^12^ ^]^ we also examined the impact of *Rfk* knockdown on the homeostatic and inflammatory genes. The results demonstrate that Rfk siRNA suppressed *Il‐1b* (FC = 0.758, *p* < 0.0001), *Il‐6* (FC = 0.598, *p* < 0.0001), *Tnfa* (FC = 0.622, *p* < 0.0001), *Ifng* (FC = 0.537, *p* = 0.0021), *Cx3cr1* (FC = 0.370, *p* = 0.0057), *Tmem119* (FC = 0.266, *p* < 0.0001) and *Csf1r* (FC = 0.514, *p* < 0.0001) mRNA expression, while increasing *P2ry12* (FC = 1.371, *p* < 0.0193) mRNA expression in LPS‐treated cells (Figure 2E,F). Furthermore, *Rfk* knockdown reduced LPS‐induced IL‐1*β*, IL‐6 and TNF‐*α* protein expression in cultured supernatants (IL‐1*β*: FC = 0.834, *p* = 0.0005; IL‐6: FC = 0.842, *p* = 0.0220; TNF‐*α*: FC = 0.776, *p* = 0.0160) (Figure 2G). We further verified the anti‐inflammatory effects of Rfk siRNA in LPS‐treated primary microglia (Figure 2H–L, Figure S5, Supporting Information). These results indicate that RFK expression is increased in LPS‐induced in vitro and in vivo brain inflammation models, and that the increased RFK expression contributes to LPS‐induced TNFR1/NF‐*κ*B signaling activation.

### 2.2 FMN, but not FAD, Inhibits LPS‐Induced Inflammatory Response

**Figure 3:**
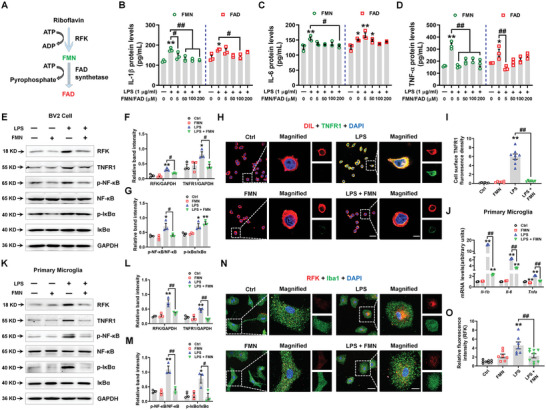
FMN supplementation suppresses LPS‐induced inflammation in BV2 cells and primary microglia. A) Schematic model of the riboflavin biosynthesis metabolic pathway. RFK, riboflavin kinase; FMN, flavin mononucleotide; FAD, flavin adenine dinucleotide. B‐D) The supernatant levels of IL‐1*β*, IL‐6, and TNF‐*α* in LPS‐treated BV2 cell cultures with different doses of FMN or FAD *n* = 3 per group. E–G) Representative blots and quantification of RFK, TNFR1, p‐NF‐*κ*B, NF‐*κ*B, p‐I*κ*B*α*, and I*κ*B*α* expression levels in LPS‐treated BV2 cells with 200 µM FMN. *n* = 3 per group. H,I) Immunofluorescence staining and quantification of TNFR1 with the membrane dye DIL in LPS‐treated BV2 cells with FMN *n* = 6–8. Scale bars, 20 µm. Magnified images are shown in the right columns. Scale bars, 5 µm. (J) The mRNA expression levels of *Il‐1b*, *Il‐6*, and *Tnfa* in LPS‐treated primary microglia with 200 µM FMN *n* = 3 per group. K‐M) Representative blots and quantification of RFK, TNFR1, p‐NF‐*κ*B, NF‐*κ*B, p‐I*κ*B*α* and I*κ*B*α* expression levels in LPS‐treated primary microglia with 200 µM FMN *n* = 3 per group. N,O) Immunofluorescence staining and quantification of RFK with Iba1 in LPS‐treated primary microglia with 200 µM FMN *n* = 7–9. Scale bars, 40 µm. Magnified images are shown in the right columns. Scale bars, 13 µm. Results are expressed as mean ± SEM. ^**^ *p* < 0.01, *p < 0.05 versus Ctrl; ^##^ *p* < 0.01, ^#^ *p* < 0.05 versus LPS. Statistical significance was determined using one‐way ANOVA and Tukey's tests for *post hoc* comparisons.

Riboflavin (vitamin B2) is metabolized to FMN and FAD via reactions catalyzed consecutively by RFK and FAD synthetase (FADS) (**Figure** **3**A). FMN and FAD then bind tightly or covalently to flavoenzymes and participate in the regulation of a range of redox reactions.^[^ ^13^ ^]^ To examine the effects of FMN and FAD on RFK expression, we injected FMN or FAD into untreated mice for five weeks (Figure S6A, Supporting Information). The results suggest that FMN, but not FAD, decreases RFK protein and mRNA expression in the hippocampus (Figure S6B‐D, Supporting Information). FMN also reduced *Il‐1b*, *Tnfa*, *Ifng*, and *Csf1r* mRNA expression in the hippocampus, while FAD showed no obvious effects on their expression levels (Figure S6E‐H, Supporting Information).

Next, we evaluated the effects of FMN and FAD on the LPS‐induced inflammatory reaction in BV2 cells and primary microglia. Different doses of FMN inhibited the LPS‐induced expression of *Il‐1b*, *Il‐6*, *Tnfa*, and *Ifng*; in contrast, FAD had no detectable anti‐inflammatory effects in LPS‐treated BV2 cells (Figure S6I‐L, Supporting Information). FMN also decreased LPS‐induced IL‐1*β*, IL‐6, and TNF‐*α* expression in the culture supernatant of BV2 cells, while FAD reduced IL‐1*β* and TNF‐*α* expression at selected concentrations (Figure 3B–D). We then chose 200 µM FMN to test its effects on microglial TNFR1/NF‐*κ*B signaling. Here, FMN downregulated the LPS‐enhanced RFK and TNFR1 expression (RFK: FC = 0.582, *p* = 0.0263; TNFR1: FC = 0.517, *p* = 0.0476), as well as the phosphorylation of NF‐*κ*B in BV2 cells (FC = 0.556, *p* = 0.0239) (Figure 3E–G). Immunostaining results verified the decreased TNFR1 expression upon FMN treatment in LPS‐treated BV2 cells (FC = 0.086, *p* < 0.0001) (Figure 3H,I). Additionally, FMN reversed the LPS‐increased BV2 cell viability, suggesting that it may suppress LPS‐induced microglial proliferation (Figure S7, Supporting Information). FMN also reduced *Il‐1b* (FC = 0.144, *p* < 0.0001), *Il‐6* (FC = 0.506, *p* < 0.0001), and *Tnfa* (FC = 0.518, *p* < 0.0001) mRNA expression and suppressed the expression of RFK (FC = 0.488, *p* = 0.0037) and TNFR1/NF‐*κ*B signaling pathway in primary microglia (TNFR1: FC = 0.283, *p* = 0.0008; p‐NF‐*κ*B: FC = 0.331, *p* = 0.0004; p‐I*κ*B*α*: FC = 0.205, *p* = 0.0143) (Figure 3J–M). Immunostaining results verified the reduced RFK expression upon FMN treatment in LPS‐treated microglia (FC = 0.493, *p* = 0.0089) (Figure 3N,O). These results indicate that FMN suppresses LPS‐induced pro‐inflammatory effects via inhibiting RFK in microglia.

### 2.3 The Inhibitory Effects of FMN on RFK are Mediated by Regulation of KMT2B

**Figure 4:**
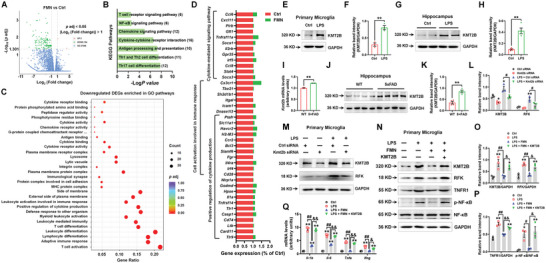
FMN regulates RFK expression via KMT2B. Wild‐type mice were intraperitoneally injected with 40 mg/kg FMN for 5 weeks, and the hippocampal samples were subjected for RNA‐seq. A) Volcano plot showing the DEGs between FMN and Ctrl mice. B,C) Representative KEGG and GO pathways enriched by downregulated DEGs between FMN and Ctrl mice. D) DEGs enriched in the “Cytokine‐mediated signaling pathway”, “Cell activation involved in immune response”, and “Positive regulation of cytokine production”. E,F) Representative blots and quantification showing KMT2B expression levels in Control and LPS‐treated primary microglia *n* = 3 per group. G,H) Representative blots and quantification showing KMT2B expression levels in the hippocampus of Control mice and the LPS mouse model *n* = 4 per group. (I) *Kmt2b* mRNA expression levels in the hippocampus of WT mice and the 5xFAD mouse model. *n* = 3 per group. J,K) Representative blots and quantification showing KMT2B expression levels in the hippocampus of WT mice and the 5xFAD mouse model *n* = 4 per group. L,M) Representative blots and quantification showing KMT2B and RFK expression levels in LPS‐treated primary microglia with Control siRNA or Kmt2b siRNA *n* = 3 per group. N–P) Representative blots and quantification of KMT2B, RFK, TNFR1, p‐NF‐*κ*B, and NF‐*κ*B expression levels in LPS‐treated primary microglia with FMN and KMT2B recombinant protein *n* = 3 per group. Q) The mRNA expression levels of *Il‐1b*, *Il‐6*, *Tnfa*, and *Ifng* in LPS‐treated primary microglia with FMN and KMT2B recombinant protein *n* = 3 per group. Results are expressed as mean ± SEM. ^**^ *p* < 0.01, *p < 0.05 versus Ctrl or Ctrl siRNA; ^##^ *p* < 0.01, ^#^ *p* < 0.05 versus LPS or LPS + Ctrl siRNA; ^&&^ *p* < 0.01, ^&^ *p* < 0.05 versus LPS + FMN. Statistical significance was determined using Student's *t*‐test (for E‐K) and one‐way ANOVA and Tukey's tests for *post hoc* comparisons (for L‐Q).

We further explored the anti‐inflammatory effects of FMN, including whether these effects rely on regulation of RFK. First, we employed RNA‐sequencing (RNA‐seq) to identify the transcriptional profiles in the hippocampus of FMN treatment (40 mg kg^−1^ dosage was chosen because it dramatically reduced RFK expression). Consistent with our in vivo results (Figure S6E–H), RNA‐seq data uncovered 154 downregulated differentially expressed genes (DEGs), most of which enriched in inflammation‐related pathways, such as “Cytokine‐cytokine receptor interaction”, “Chemokine signaling pathway”, and “NF‐*κ*B signaling pathway” (**Figure** **4**A–C, Figure S8, Supporting Information). Representative DEGs are listed in Figure 4D. These results confirmed the anti‐inflammatory effects of FMN, which we also observed in vitro.

Next, we sought to determine the mechanism by which FMN could inhibit RFK expression. Recently, RFK was shown to be epigenetically regulated at the transcriptional level by a histone H3 lysine 4 (H3K4) methyltransferase, KMT2B.^[^ ^14^ ^]^ We, therefore, examined whether FMN regulates RFK via KMT2B. Consistent with this possibility, we found increased expression levels of KMT2B in LPS‐treated microglia (FC = 2.663, *p* = 0.0032), and in the hippocampus of LPS‐treated mice (mRNA level: FC = 1.603, *p* < 0.0001; protein level: FC = 4.505, *p* = 0.0008) and 5xFAD mice (mRNA level: FC = 1.214, *p* = 0.0002; protein level: FC = 2.408, *p* = 0.0010) (Figure 4E–K, Figure S9A, Supporting Information). Next, using siRNA targeting *Kmt2b* (siRNA‐3, Figure S9B–D, Supporting Information), we determined that *Kmt2b* knockdown abolished LPS‐induced RFK expression (FC = 0.468, *p* = 0.0255) (Figure 4L,M). Additionally, FMN reduced KMT2B expression in LPS‐treated primary microglia and in the hippocampi of untreated mice (Figure S9E‐H, Supporting Information). Importantly, we found that recombinant KMT2B protein successfully prevented FMN's effects on RFK, TNFR1/NF‐*κ*B signaling, and pro‐inflammatory cytokines in LPS‐treated primary microglia (Figure 4N–Q, Figure S9I,J, Supporting Information).

Thus, these results indicate that FMN inhibits the LPS‐induced inflammatory response via regulation of RFK/KMT2B, suggesting that FMN may comprise a novel and promising candidate for therapeutic anti‐inflammation in neuroinflammatory diseases.

### 2.4 Hippocampal *Rfk* Knockdown Rescues Cognitive Impairment and the Pro‐Inflammatory Response

**Figure 5:**
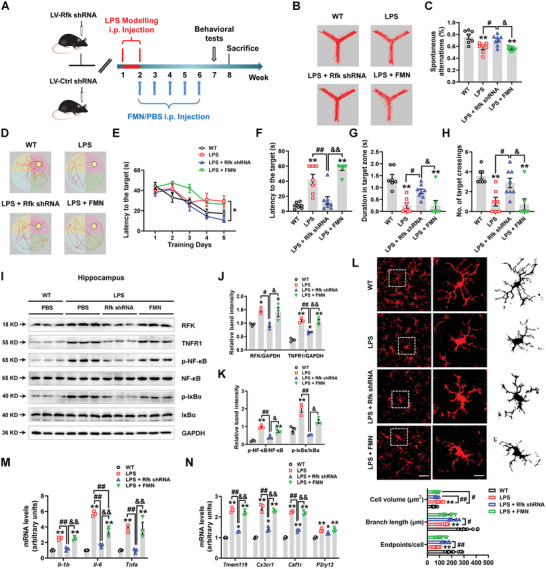
Hippocampal *Rfk* knockdown attenuates LPS‐induced cognitive impairment and inflammatory response. A) Experimental design for *Rfk* knockdown and FMN administration in the LPS‐induced mouse model. B) Travel path tracings of mice in the Y‐maze test. C) Spontaneous alterations of LPS mice treated with LV‐Rfk shRNA or FMN in the Y‐maze. D) Representative path tracings in each quadrant during the probe trial. E‐H) The escape latency over a five‐day training course (panel E), latency in the probe test (panel F), time spent in the target zone (panel G), and number of target crossings (panel H) in the Morris water maze *n* = 7–8. I‐K) Representative blots and quantification of RFK, TNFR1, p‐NF‐*κ*B, NF‐*κ*B, p‐I*κ*B*α* and I*κ*B*α* expression levels in the hippocampus of LPS mice treated with LV‐Rfk shRNA or FMN *n* = 3 per group. L) Immunofluorescence staining and quantification of Iba1‐positive cells in the hippocampus of LPS mice treated with LV‐Rfk shRNA or FMN. Scale bars, 40 µm. Magnified images and skeletal diagrams of Iba1‐positive cells are shown to the right of each staining image. Scale bars, 10 µm *n* = 8–9. M,N) The mRNA expression levels of *Il‐1b*, *Il‐6*, *Tnfa*, *Tmem119*, *Cx3cr1*, *Csf1r*, and *P2ry12* in the hippocampus of LPS mice treated with LV‐Rfk shRNA or FMN *n* = 3 per group. Results are expressed as mean ± SEM. ^**^ *p* < 0.01, *p < 0.05 versus WT; ^##^ *p* < 0.01, ^#^ *p* < 0.05 versus LPS; ^&&^ *p* < 0.01, ^&^ *p* < 0.05 versus LPS + Rfk shRNA. Statistical significance was determined using one‐way ANOVA and Tukey's tests for *post hoc* comparisons.

We further compared the effects of *Rfk* knockdown with FMN supplementation in LPS‐treated mice (**Figure** **5**A). Here, we found that *Rfk* knockdown promoted recovery of the spontaneous alterations in the Y maze, as compared with the LPS and LPS + FMN groups (FC = 1.218, *p* = 0.0377 and FC = 1.260, *p* = 0.0136) (Figure 5B,C). Moreover, *Rfk* knockdown decreased the latency to the target during five training days in the Morris water maze, as compared with the LPS and LPS + FMN groups (Figure 5D,E). In the probe test, *Rfk* knockdown reduced the latency to the target (FC = 0.341, *p* = 0.0011 and FC = 0.253, *p* < 0.0001), and increased the duration time in the target zone (FC = 3.408, *p* = 0.0369 and FC = 3.331, *p* = 0.0393) and number of target crossings (FC = 2.875, *p* = 0.0332 and FC = 4.025, *p* = 0.0118), as compared with LPS and LPS + FMN groups (Figure 5F–H).

*Rfk* knockdown also inhibited TNFR1/NF‐*κ*B signaling in the hippocampus, as compared with the LPS and LPS + FMN groups (Figure 5I–K). Furthermore, *Rfk* knockdown suppressed microglia activation (Figure 5L), as well as inhibited the mRNA expression of *Il‐1b* (FC = 0.461, *p* = 0.0001 and FC = 0.469, *p* = 0.0002), *Il‐6* (FC = 0.289, *p* < 0.0001 and FC = 0.476, *p* = 0.0016), *Tnfa* (FC = 0.258, *p* = 0.0067 and FC = 0.249, *p* = 0.0050), *Tmem119* (FC = 0.549, *p* < 0.0001 and FC = 0.586, *p* < 0.0001), *Cx3cr1* (FC = 0.573, *p* < 0.0001 and FC = 0.618, *p* = 0.0001) and *Csf1r* (FC = 0.597, *p* < 0.0001 and FC = 0.614, *p* < 0.0001), as compared with the LPS and LPS + FMN groups (Figure 5M,N). These data suggest that *Rfk* knockdown, but not direct FMN supplementation, attenuates LPS‐induced pro‐inflammatory effects and cognitive impairment.

### 2.5 Characterization, Brain Targeting, and Biodistribution of MNPs@FMN

**Figure 6:**
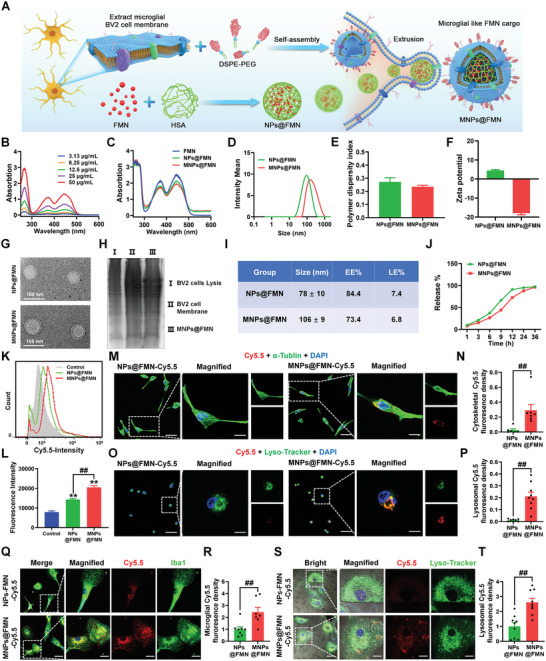
Biological effects of MNPs@FMN on microglial BV2 cells. A) Schematic illustration of the preparation of MNPs@FMN. B) UV–vis absorbance of FMN. C) UV–vis absorbance of FMN, NPs@FMN and MNPs@FMN. D) Particle size analysis of NPs@FMN and MNPs@FMN. E) polydispersity index analysis of NPs@FMN and MNPs@FMN *n* = 3 per group. F) Zeta potential of NPs@FMN and MNPs@FMN *n* = 3 per group. G) Representative transmission electron microscopy images. Scale bars, 100 nm. H) Representative blots of BV2 cell lysis, BV2 cell membrane, and MNPs@FMN. I) EE% and LE% NPs@FMN and MNPs@FMN were determined by UV spectrophotometer. J) The drug release kinetics of NPs@FMN and MNPs@FMN *n* = 3 per group. K,L) Flow cytometry analysis of cellular uptake of NPs@FMN and MNPs@FMN *n* = 3 per group. M,N) Representative images and quantitative fluorescence analysis of cytoskeletal *α*‐Tubulin in BV2 cells after 3 h incubation with Cy5.5‐labelled NPs@FMN and MNPs@FMN *n* = 6–7. (O and P) Representative images and quantitative fluorescence analysis of lysosomal tracker (Lyso‐tracker) in BV2 cells after 3 h incubation with Cy5.5‐labelled NPs@FMN and MNPs@FMN *n* = 8–9. Scale bars, 50 µm. Magnified images are shown in the right columns of panels M and O Scale bars, 10 µm. Q,R) Representative images and quantitative fluorescence analysis of Iba1 in primary microglia after 3 h incubation with Cy5.5‐labelled NPs@FMN and MNPs@FMN *n* = 7–8. S,T) Representative images and quantitative fluorescence analysis of Lyso‐tracker in primary microglia after 3 h incubation with Cy5.5‐labelled NPs@FMN and MNPs@FMN *n* = 9 per group. Scale bars, 40 µm. Magnified images are shown in the right columns of panels Q and S. Scale bars, 13 µm. Results are expressed as mean ± SEM. ^**^ *p* < 0.01 versus Control; ^##^ *p* < 0.01 versus NPs@FMN. Statistical significance was determined using one‐way ANOVA and Tukey's tests for *post hoc* comparisons (for panels K and L) and the Student's *t*‐test (for panels N, P, R, and T).

Because direct FMN supplementation did not recapture the anti‐inflammatory effects of RFK, we speculated that the BBB, drug duration or cell targeting specificity may hinder its effects. Therefore, we developed an effective strategy for targeted FMN delivery (termed MNPs@FMN) via the following steps: first, we extracted microglial BV2 cell membranes; second, we prepared FMN‐encapsulated HSA nanoparticles as the core; lastly, we bioorthogonally attached isolated microglial membranes onto the surface of the HSA‐FMN nanoparticles (**Figure** **6**A). We used the ultraviolet absorption at 450 nm to draw a standard curve of FMN (Figure 6B, Figure S10A, Supporting Information). FMN, NPs@FMN, and MNPs@FMN have similar absorption peaks (Figure 6C). Furthermore, the average hydrodynamic diameter of the NPs@FMN increased from 78 nm to 106 nm with a polydispersity index of 0.285 and 0.229 after they were coated with the microglial membranes (Figure 6D,E). The results from *ζ*‐potential, transmission electron microscopy images and sodium dodecyl sulfate polyacrylamide gel electrophoresis (SDS‐PAGE) demonstrated that the MNPs@FMN nanoparticles had been successfully engulfed by the BV2 microglial membrane (Figure 6F–H). The drug encapsulation efficiency (EE)%, loading efficiency (LE) and release behaviors suggest that MNPs@FMN is released more slowly and thus may be long‐lasting (Figure 6I,J). Flow cytometry results showed that MNPs@FMN exhibited a greater efficiency in cellular uptake than NPs@FMN (Figure 6K,L). Moreover, these results were verified by costaining with cytoskeletal *α*‐Tubulin (Figure 6M,N). Upon uptake, MNPs@FMN accumulated in the lysosomes as indicated by costaining with lysosomal tracker (Lyso‐tracker) (Figure 6O,P). Co‐staining of Cy5.5 with Iba1 or Lyso‐tracker, and evaluation of cellular Cy5.5 uptake in primary microglia further confirmed these results (Figure 6Q,T, Figure S10B,C, Supporting Information), suggesting that MNPs@FMN was degraded in the lysosome and then FMN was released.

**Figure 7:**
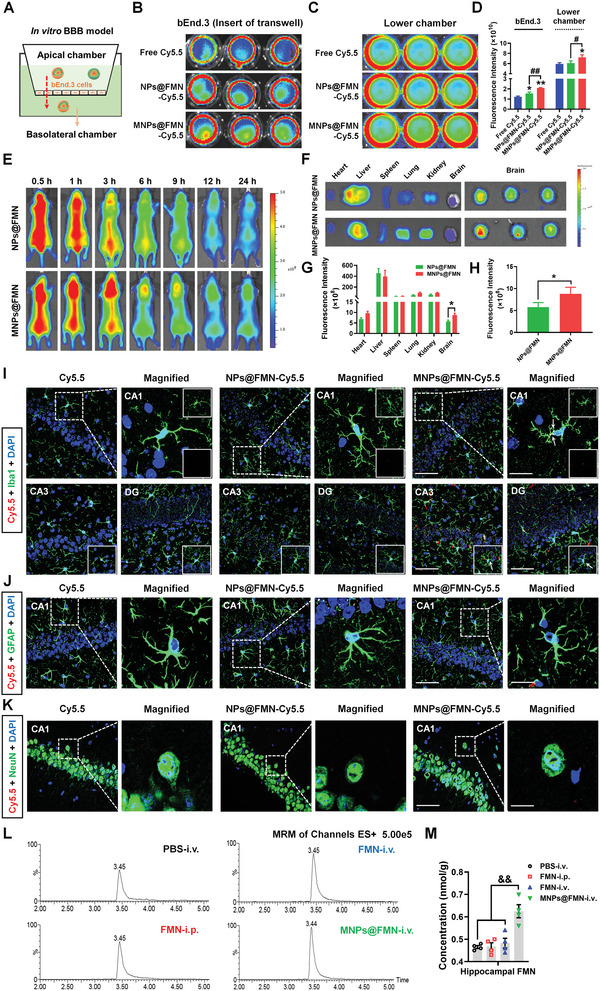
Brain‐targeted delivery of MNPs@FMN. A) Schematic diagram of the in vitro BBB model. B–D) Fluorescence images and quantitation of the bEnd.3 monolayer and basolateral chamber in the transwell after 3 h incubation with Cy5.5, NPs@FMN‐Cy5.5, or MNPs@FMN‐Cy5.5 *n* = 3 per group. E) Real time fluorescence imaging of mice after intravenously injection of Cy5.5‐labelled NPs@FMN or MNPs@FMN. F–H) Ex vivo imaging and corresponding fluorescence analysis of sacrificed tissues (heart, liver, spleen, lung, kidney, and brain) after intravenously injection of Cy5.5‐labelled NPs@FMN or MNPs@FMN *n* = 3 per group. I) Representative images of Iba1 staining at 6 h postinjection in hippocampal CA1, CA3, and DG areas derived from mice treated with Cy5.5, NPs@FMN‐Cy5.5, or MNPs@FMN‐Cy5.5. White arrows in the enlarged details of the right column show the presence of nanoparticles in microglia. J,K) Representative images of GFAP and NeuN staining at 6 h postinjection in hippocampi derived from mice treated with Cy5.5, NPs@FMN‐Cy5.5, or MNPs@FMN‐Cy5.5. Scale bars, 50 µm. Magnified images are shown in the right column of panels I‐K. Scale bars, 10 µm. (L) Mice were subjected to intraperitoneal delivery of free FMN, or intravenous delivery of PBS, free FMN, or MNPs@FMN. After 6 h, hippocampal samples were collected for UPLC‐MS/MS analysis. M) Quantification of FMN concentrations in the hippocampus *n* = 4 per group. Results are expressed as mean ± SEM. ^**^ *p* < 0.01, *p < 0.05 versus Free Cy5.5; ^##^ *p* < 0.01 versus NPs@FMN‐Cy5.5; ^&&^ *p* < 0.01 versus MNPs@FMN. Statistical significance was determined using one‐way ANOVA and Tukey's tests for *post hoc* comparisons.

The in vitro BBB permeability of MNPs@FMN results show that the fluorescence signals in the bEND.3 cell monolayer (insert) and in the lower chamber were higher after incubation with MNPs@ FMN‐Cy5.5 than after incubation with NPs@FMN‐Cy5.5, suggesting that MNPs@FMN has greater ability to cross the BBB in vitro (**Figure** **7**A–D). In vivo fluorescence imaging and ex vivo results also indicated that the brain distribution of MNPs@FMN compared with NPs@FMN‐Cy5.5 was earlier and prolonged (Figure 7E–H), providing additional physiological relevance to these findings. MNPs@FMN was obviously accumulated in the microglia rather than in neuron or astrocytes in the CA1, CA3, and DG regions of the hippocampus (Figure 7I–K). We also found much higher FMN concentration in the hippocampus after intravenous delivery of MNPs@FMN than intraperitoneal and intravenous delivery of free FMN (FC = 1.337, *p* = 0.0007 and FC = 1.295, *p* = 0.0015) purified using the ultra‐performance liquid chromatography‐tandem mass spectrometry (UPLC‐MS/MS) method (Figure 7L,M). These results suggest that MNPs@FMN has better FMN delivery efficiency.

### 2.6 MNPs@FMN Ameliorates LPS‐Induced Cognitive Deficits, Dysfunctional Synaptic Plasticity, and Inflammatory Response

**Figure 8:**
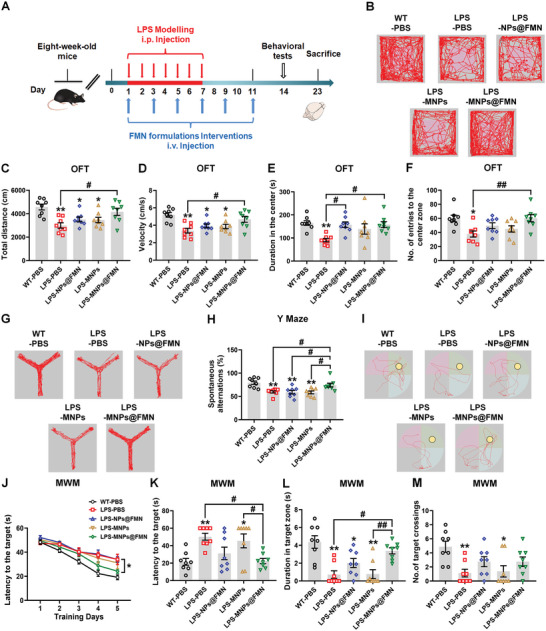
MNPs@FMN ameliorates cognitive dysfunction in the LPS‐induced mouse model. A) Experimental design for MNPs@FMN administration in the LPS‐induced mouse model. B) Travel tracings of mice in the open field. C‐F) Total distance traveled, movement speed, time spent in the center, and number of entries to the center zone in the open field of LPS mice treated with NPs@FMN, MNPs, or MNPs@FMN. G) Travel path tracings of mice in the Y‐maze test. H) Spontaneous alterations of LPS mice treated with NPs@FMN, MNPs, or MNPs@FMN in the Y maze. I) Representative path tracings in each quadrant during the probe trial. J–M) The escape latency over a five‐day training course (panel J), latency in the probe test (panel K), time spent in the target zone (panel L), and number of target crossings (panel M) in the Morris water maze *n* = 8 per group. Results are expressed as mean ± SEM. ^**^ *p* < 0.01, *p < 0.05 versus WT‐PBS; ^##^ *p* < 0.01 versus LPS‐MNPs@FMN. Statistical significance was determined using one‐way ANOVA and Tukey's tests for *post hoc* comparisons.

To evaluate whether microglial targeted delivery of FMN improves inflammation‐based cognitive dysfunction, we evaluated MNPs@FMN intervention in the LPS‐induced mouse model. Equivalent molecular doses of NPs@FMN, MNPs@FMN, MNPs, or the control vehicle were administered intravenously in the LPS‐induced model every other day for 11 days. Three days later, behavioral tests were performed to evaluate the exploratory activity and cognitive function of the mice (**Figure** **8**A). The results of the OFT suggest that MNPs@FMN significantly increased the total travelled distance (FC = 1.390, *p* = 0.0147), movement speed (FC = 1.386, *p* = 0.0139), duration in the center zone (FC = 1.738, *p* = 0.0128) and number of entries to the center zone (FC = 1.596, *p* = 0.0097) in LPS‐induced mice (Figure 8B–F). By comparison, NPs@FMN only prolonged the duration of LPS‐induced mice in the center zone (FC = 1.735, *p* = 0.0134) (Figure 8E). MNPs@FMN also restored spontaneous alterations in the Y maze, as compared with the LPS‐PBS, LPS‐NPs@FMN, and LPS‐MNPs groups (Figure 8G,H), while the number of arm entries showed no obvious differences among these five groups (Figure S11A, Supporting Information). Consistently, MNPs@FMN also decreased the latency to the target over five training days and the probe test of the Morris water maze, as compared with the latency values for the LPS‐PBS and LPS‐MNPs groups (Figure 8J,K). Moreover, MNPs@FMN increased the duration time in the target zone compared with the duration of the LPS‐PBS (FC = 4.688, *p* = 0.0063) and LPS‐MNPs (FC = 4.360, *p* = 0.0078) groups (Figure 8L), while NPs@FMN, MNPs, and MNPs@FMN showed no obvious effects on the number of target crossings, travelled distance and swim speed compared with these measurements for the LPS group (Figure 8M, Figure S11B,C, Supporting Information).

**Figure 9:**
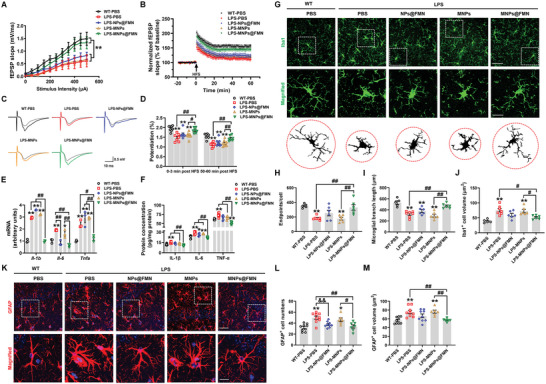
MNPs@FMN improves dysfunctional synaptic plasticity and inflammatory response in LPS‐treated mice. A) Input‐output relations are generated by stimulating the SCs and recording in CA1 stratum radiatum *n* = 7–10. (B) LTP at the SC‐CA1 synapses was recorded in the hippocampuses of LPS mice treated with NPs@FMN, MNPs, or MNPs@FMN. C) The representative traces of fEPSP recordings of responses before and 50 min after high‐frequency stimulation (HFS; arrow). D) Quantitative analysis of LTP data in B. The level of fEPSP potentiation was determined at a mean of 0–3 min and 50–60 min after high‐frequency stimulation *n* = 7–9. E) The mRNA expression levels of *Il‐1b*, *Il‐6*, and *Tnfa* in the hippocampi of LPS mice treated with NPs@FMN, MNPs, or MNPs@FMN *n* = 3 per group. F) The protein expression levels of IL‐1*β*, IL‐6, and TNF‐*α* in the hippocampi of LPS mice treated with NPs@FMN, MNPs, or MNPs@FMN *n* = 6 per group. G) Immunofluorescence staining of Iba1‐positive cells in the CA1 of hippocampi of LPS mice treated with NPs@FMN, MNPs, or MNPs@FMN. Scale bars, 40 µm. Magnified images are shown in the middle column, and skeletal diagrams of Iba1‐positive cells are shown in the bottom of panel G. Scale bars, 10 µm. H‐J) Quantification of endpoint voxels, branch length, and volume of Iba1‐positive cells *n* = 5–7. K) Immunofluorescence staining of GFAP‐positive cells in the hippocampi of LPS mice treated with NPs@FMN, MNPs, or MNPs@FMN. Scale bars, 40 µm. Magnified images are shown in the bottom of panel K Scale bars, 10 µm. L,M) Quantification of the cell numbers and cell volume of GFAP‐positive cells in the CA1 of the hippocampus *n* = 6–9. Results are expressed as mean ± SEM. ^**^ *p* < 0.01, *p < 0.05 versus WT‐PBS; ^##^ *p* < 0.01, ^#^ *p* < 0.05 versus LPS‐MNPs@FMN; ^&&^ *p* < 0.01 versus LPS‐PBS. Statistical significance was determined using one‐way ANOVA and Tukey's tests for *post hoc* comparisons.

Hippocampal synaptic plasticity is essential to learning and memory.^[^ ^15^ ^]^ Therefore, we examined the effects of MNPs@FMN on the long‐term potentiation (LTP) in the hippocampus. We first detected the synaptic function in the Schaffer collateral pathway (SC‐CA1) in hippocampal slices and recorded fEPSPs in the CA1 stratum radiatum by stimulating the SC/commissural pathway at various intensities. MNPs@FMN increased fEPSP slopes in CA1 hippocampal slices over those associated with LPS‐PBS, LPS‐MNPs, and LPS‐NPs@FMN (**Figure** **9**A). Then, we detected the LTP at the SC‐CA1 synapses. MNPs@FMN promoted LTP amplitude at 0–3 min and 50–60 min after LTP induction relative to the LPS‐PBS (0–3 min: FC = 1.253, *p* = 0.0013; 50–60 min: FC = 1.291, *p* = 0.0016) and LPS‐MNPs (0–3 min: FC = 1.161, *p* = 0.0286; 50–60 min: FC = 1.217, *p* = 0.0099) groups (Figure 9B–D). These results indicate that MNPs@FMN restores hippocampal synaptic plasticity in LPS‐induced mice with cognitive decline.

Because LPS‐induced cognitive deficits are initiated by the inflammatory response, we further examined the effects of MNPs@FMN on inflammation. As compared with the LPS‐PBS and LPS‐MNPs groups, the LPS‐MNPs@FMN group displayed significantly lower *Il‐1b* (FC = 0.332, *p* < 0.0001; FC = 0.302, *p* < 0.0001), *Il‐6* (FC = 0.328, *p* = 0.0001; FC = 0.391, *p* = 0.0012) and *Tnfa* (FC = 0.509, *p* = 0.0130; FC = 0.419, *p* = 0.0010) mRNA expression, as well as IL‐1*β* (FC = 0.707, *p* < 0.0001; FC = 0.779, *p* = 0.0011), IL‐6 (FC = 0.760, *p* < 0.0001; FC = 0.793, *p* < 0.0001) and TNF‐*α* (FC = 0.704, *p* < 0.0001; FC = 0.777, *p* < 0.0001) protein expression in the hippocampus; furthermore, the reduction in *Il‐1b* (FC = 0.271, *p* < 0.0001) and *Tnfa* (FC = 0.421, *p* = 0.0010) mRNA expression and IL‐1*β* (FC = 0.784, *p* = 0.0016), IL‐6 (FC = 0.844, *p* = 0.0040) and TNF‐*α* (FC = 0.779, *p* = 0.0001) protein expression was much more obvious for the LPS‐MNPs@FMN group than for the LPS‐NPs@FMN group (Figure 9E,F). Furthermore, as compared with the LPS‐PBS and LPS‐MNPs groups, the MNPs@FMN group displayed increased endpoint voxels (FC = 1.813, *p* = 0.0034; FC = 1.867, *p* = 0.0035) and process complexity (FC = 1.452, *p* = 0.0013; FC = 1.590, *p* = 0.0002) and a lower volume (FC = 0.686, *p* = 0.0102; FC = 0.713, *p* = 0.0367) for Iba1‐positive cells (Figure 9G–J). MNPs@FMN also suppressed astroglial activation in the CA1 of the hippocampus of LPS‐treated mice compared with LPS‐PBS and LPS‐MNPs (cell numbers: FC = 0.678, *p* = 0.0015; FC = 0.736, *p* = 0.0482; cell volume: FC = 0.756, *p* = 0.0052; FC = 0.748, *p* = 0.0091) (Figure 9K–M).

Because our observations suggest that MNPs@FMN also localizes, in part, to the liver, lung, and kidney of mice after intravenous administration, we further examined the potential adverse effects of MNPs@FMN on non‐targeted organs. However, we did not detect pathological changes in the liver, heart, kidney, or lung as evaluated by H&E staining after MNPs@FMN administration (Figure S12, Supporting Information). Moreover, there were no detectable alterations in serum biochemical indicators of liver or kidney function after NPs@FMN and MNPs@FMN administration (Table S1, Supporting Information). These results suggest that MNPs@FMN restores cognitive function and synaptic plasticity in LPS‐induced mice by suppressing the inflammatory response, with no obvious side effects.

### 2.7 Transcriptome Mechanism of MNPs@FMN in Improving Inflammation‐Based Cognitive Dysfunction

**Figure 10:**
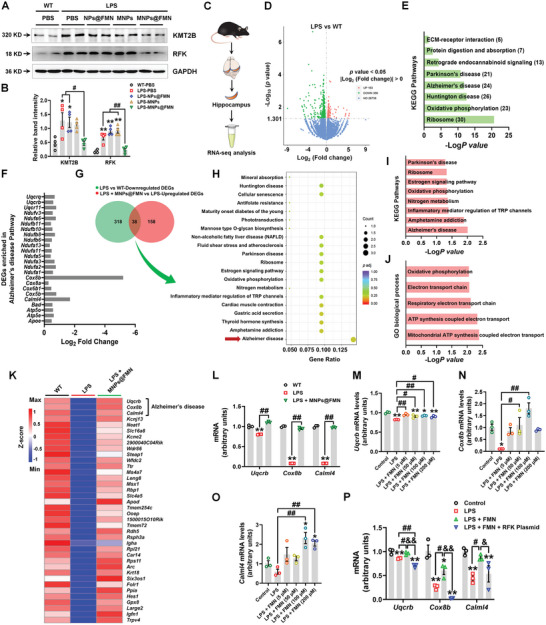
RNA‐seq analysis of MNPs@FMN treatment in the LPS‐induced mouse model. A,B) Representative blots and quantification showing KMT2B and RFK expression levels in the hippocampi of LPS mice treated with NPs@FMN, MNPs, or MNPs@FMN, *n* = 4 per group. C) Schematic illustration of RNA‐seq. D) Volcano plot showing the DEGs between the LPS and WT groups. E) KEGG pathways enriched by downregulated DEGs in the LPS compared with WT group. F) Downregulated DEGs in the LPS groups that are enriched in the “Alzheimer's disease” KEGG pathway. G) Venn diagram showing 38 DEGs that were downregulated by LPS and upregulated by MNPs@FMN. H) KEGG pathways are enriched by these 38 DEGs. I,J) KEGG and GO pathways enriched by upregulated DEGs in LPS + MNPs@FMN compared with LPS. K) Hierarchical clustering of the 38 LPS + MNPs@FMN reversed DEGs. L) The mRNA expression levels of *Uqcrb*, *Cox8b*, and *Calml4* in the hippocampi of the WT, LPS, and LPS + MNPs@FMN groups. *n* = 3 per group. M–O) The mRNA expression levels of *Uqcrb*, *Cox8b*, and *Calml4* upon treatment with different concentrations of FMN in LPS‐treated BV2 cells *n* = 3 per group. P) The mRNA expression levels of *Uqcrb*, *Cox8b*, and *Calml4* upon FMN and RFK plasmid treatment in LPS‐treated BV2 cells. *n* = 3 per group. Results are expressed as mean ± SEM. ^**^ *p* < 0.01, *p < 0.05 versus WT or Control; ^##^ *p* < 0.01, ^#^ *p* < 0.05 versus LPS‐MNPs@FMN or LPS. Statistical significance was determined using one‐way ANOVA and Tukey's tests for *post hoc* comparisons.

Because our results indicated that FMN may reduce the pro‐inflammatory response via feedback regulation of RFK, we further evaluated the effect of MNPs@FMN on hippocampal RFK levels. Western blotting and PCR assays indicate that MNPs@FMN decreased LPS‐induced hippocampal KMT2B and RFK expression (**Figure** **10**A,B, Figure S13, Supporting Information). We further examined the underlying mechanisms of MNPs@FMN in improving LPS‐induced cognitive deficits via RNA‐seq (Figure 10C). PCA score plots revealed a distinct separation of components in the WT, LPS, and LPS + MNPs@FMN groups (Figure S14A, Supporting Information), for which the gene expression and FPKM distributions were similar (Figure S14B–D, Supporting Information). Furthermore, volcano plot revealed 519 DEGs between the LPS and WT groups, and the downregulated DEGs were enriched in biologically meaningful Kyoto Encyclopedia of Genes and Genomes (KEGG) pathways, such as “Ribosome,” “Oxidative phosphorylation,” “Huntington disease,” “Alzheimer's disease,” and “Parkinson's disease” (Figure 10D,E). The downregulated DEGs that were enriched in the “Alzheimer's disease” pathway are shown in Figure 10F.

We further identified 38 DEGs that were decreased by LPS and reversed by MNPs@FMN treatment, and indicated their enriched signaling pathways (Figure 10G–J, Figure S15, Supporting Information). Among the MNPs@FMN‐altered DEGs, ubiquinol cytochrome c reductase binding protein (*Uqcrb*), cytochrome C oxidase subunit 8b (*Cox8b*), and calmodulin‐like protein 4 (*Calml4*) were enriched in the “Alzheimer's disease” pathway (Figure 10K). We verified the expression of these three genes in the hippocampus by qRT‐PCR (Figure 10L). Furthermore, we confirmed that FMN reversed the effect of LPS in decreasing *Uqcrb*, *Cox8b*, and *Calml4* expression in microglial BV2 cells, and that RFK overexpression abolished the effects of FMN (Figure 10M–P). Thus, these results reveal a molecular mechanism underlying MNPs@FMN‐improved cognitive function in LPS‐induced mice.

### 2.8 MNPs@FMN Improves Cognitive Function in the 5xFAD Mouse Model of AD

**Figure 11:**
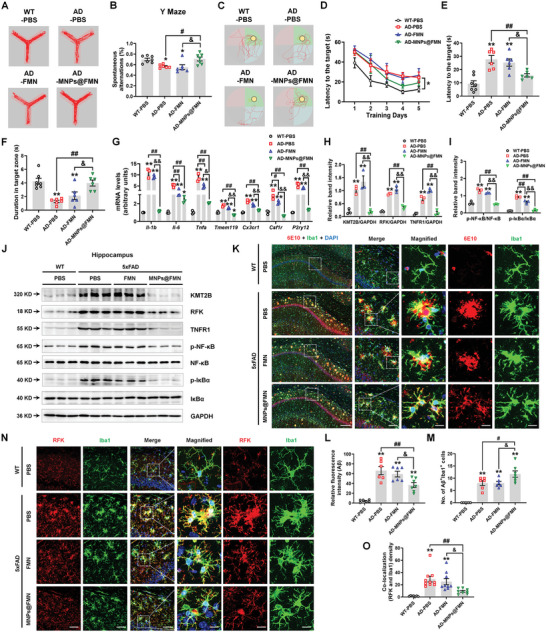
MNPs@FMN improves cognitive function and reduces pro‐inflammatory response in the 5xFAD mouse model. A) Travel path tracings of mice in the Y maze test. B) Spontaneous alterations of 5xFAD mouse intravenous delivery of PBS, free FMN, or MNPs@FMN in the Y‐maze. C) Representative path tracings in each quadrant during the probe trial. D–F) The escape latency over a five‐day training course (panel D), latency in the probe test (panel E), and time spent in the target zone (panel F) in the Morris water maze. *n* = 6–7. G) The mRNA expression levels of *Il‐1b*, *Il‐6*, *Tnfa*, *Tmem119*, *Cx3cr1*, *Csf1r*, and *P2ry12* in the hippocampi of 5xFAD mice after intravenous delivery of PBS, free FMN or MNPs@FMN. n = 3 per group. H–J) Representative blots and quantification of KMT2B, RFK, TNFR1, p‐NF‐*κ*B, NF‐*κ*B, p‐I*κ*B*α* and I*κ*B*α* expression levels in the hippocampi of 5xFAD mice after intravenous delivery of PBS, free FMN or MNPs@FMN. *n* = 3 per group. K) Immunofluorescence staining of A*β* plaque (6E10) with Iba1 in the CA1 of hippocampi of 5xFAD mice after intravenous delivery of PBS, free FMN, or MNPs@FMN. Scale bars, 100 µm for the left full view panel. Scale bars, 40 µm for Merge panel, and 10 µm for magnified images. L,M) Quantification of A*β* intensity and the number of microglia engulfing A*β* in the CA1 of the hippocampus *n* = 6–7. N,O) Immunofluorescence staining and quantification of RFK with Iba1 in the CA1 of hippocampi of 5xFAD mice after intravenous delivery of PBS, free FMN, or MNPs@FMN. Scale bars, 40 µm. Magnified images are shown in the right. Scale bars, 10 µm *n* = 7–10. Results are expressed as mean ± SEM. ^**^ *p* < 0.01, *p < 0.05 versus WT; ^##^ *p* < 0.01, ^#^ *p* < 0.05 versus AD; ^&&^ *p* < 0.01, ^&^ *p* < 0.05 versus AD‐FMN. Statistical significance was determined using one‐way ANOVA and Tukey's tests for *post hoc* comparisons.

Given that our RNA‐seq data revealed MNPs@FMN regulation of pathways related to AD, we compared its effects with that of intravenous delivery of free FMN in the 5xFAD mouse model of AD. MNPs@FMN increased the spontaneous alterations in the Y maze, as compared with the AD‐PBS and AD‐FMN groups (FC = 1.244, *p* = 0.0325; FC = 1.280, *p* = 0.0153) (**Figure** **11**A,B). During five days’ training in the Morris water maze, MNPs@FMN shortened the latency to the target (Figure 11C,D). In the probe test, MNPs@FMN significantly reduced the latency to the hidden target (FC = 0.564, *p* = 0.0091; FC = 0.617, *p* = 0.0430) and increased the duration in the target zone (FC = 3.098, *p* = 0.0015; FC = 1.881, *p* = 0.0312), as compared with the AD‐PBS and AD‐FMN groups (Figure 11E,F). Furthermore, MNPs@FMN suppressed the increased levels of inflammatory‐related genes (*Il‐1b*: FC = 0.104, *p* < 0.0001; FC = 0.125, *p* = 0.0001; *Il‐6*: FC = 0.466, *p* = 0.0004; FC = 0.742, *p* = 0.194; *Tnfa*: FC = 0.240, *p* = 0.0002; FC = 0.425, *p* = 0.0353; *Tmem119*: FC = 0.397, *p* < 0.0001; FC = 0.473, *p* < 0.0001; *Cx3cr1*: FC = 0.543, *p* < 0.0001; FC = 0.673, *p* = 0.0006; *Csf1r*: FC = 0.215, *p* < 0.0001; FC = 0.285, *p* < 0.0001; *P2ry12*: FC = 0.236, *p* < 0.0001; FC = 0.318, *p* = 0.0009) in the hippocampus, as compared with the AD‐PBS and AD‐FMN groups (Figure 11G). Consistently, MNPs@FMN, but not free FMN, inhibited KMT2B and RFK expression and TNFR1/NF‐*κ*B signaling in the hippocampus of the 5xFAD mouse model (Figure 11H–J). Intriguingly, MNPs@FMN reduced the A*β* plaque burden (stained by 6E10) (FC = 0.556, *p* = 0.0061; FC = 0.622, *p* = 0.0439), and increased the numbers of microglia engulfing A*β* (FC = 1.463, *p* = 0.0441; FC = 1.463, *p* = 0.0441) in the hippocampus, together with decreased microglial RFK expression in the AD model (FC = 0.321, *p* = 0.0050; FC = 0.374, *p* = 0.0385), as compared with AD‐PBS and AD‐FMN (Figure 11K–O). Additionally, the microglia assumed a less “activated” phenotype (Figure 11K–N). These findings indicate that MNPs@FMN rescues cognitive function, pro‐inflammatory response, and A*β* plaque pathology in an AD mouse model.

## 3 Discussion

Low intake of riboflavin has been correlated with the development of cognitive impairment, and studies suggest that cognitive function in middle‐aged and elderly people may be improved by dietary intake of riboflavin and other forms of vitamin B.^[^ ^16^ ^]^ Notably, the cognitive protection of riboflavin may be associated with its anti‐oxidative and anti‐inflammatory effects.^[^ ^9^ ^]^ As a key enzyme in riboflavin metabolism, RFK has been shown to couple with TNFR1 to active NADPH oxidase,^[^ ^11^ ^]^ suggesting that it may play a role in mediating the anti‐inflammatory effects of riboflavin. Here, we reported for the first time that RFK is expressed in the microglia and that it is upregulated in the cortex and hippocampus of the LPS‐induced mouse model and 5xFAD mouse model of AD, suggesting that it may be related to inflammation‐based cognitive impairment. Functionally, TNFR1 induces RFK membrane recruitment and subsequent phosphorylation of NF‐*κ*B, which leads to the transcription of pro‐inflammatory cytokines, such as IL‐1*β*, IL‐6, and TNF‐*α*. In vitro and in vivo *Rfk* knockdown attenuates the pro‐inflammatory response mediated by the TNFR1/NF‐*κ*B signaling pathway, and hippocampal *Rfk* knockdown improves the cognitive function in the LPS‐treated mouse model. Rfk mRNA expression has previously been demonstrated to be decreased in AD patients and the A*β*‐based yeast model.^[^ ^10^ ^]^ However, in our study, we observed increased RFK protein expression in the cortex and hippocampus of the LPS‐induced mouse model and 5xFAD mouse model of AD. These disparate findings may result from differences in the model systems, given that we mainly focused on murine RFK function. It is worth noting that the latter study limited its evaluation of AD patients to the cerebellum, prefrontal cortex, and visual cortex.^[^ ^10^ ^]^ Therefore, additional experimentation will be needed to further characterize the RFK regulatory patterns in different regions of the brain.

Importantly, our results provide a mechanism by which FMN may have therapeutic efficacy for neurological disorders. FMN and FAD, which are catalyzed by RFK and FADS, serve as critical mediators of riboflavin metabolism, and as cofactors for flavoenzymes, they contribute to the maintenance of normal flavoprotein functionality, with impacts on redox reactions, DNA repair, protein folding, and other physiological processes. FMN was previously reported to prevent A*β* toxicity by increasing ATP and NADH generation.^[^ ^10^ ^]^ Moreover, our lab demonstrated that FMN protects DA neurons from oxidative stress in the PD mouse model.^[^ ^17^ ^]^ In the present study, we report that FMN, but not FAD, improves inflammation‐based cognitive function by regulating the TNFR1/NF‐*κ*B pathway. While H3K4 methyltransferase KMT2B induces RFK transcription to activate the TNF‐*α*/NOX2 pathway, we also found that KMT2B is the upstream regulator of RFK, and interestingly, KMT2B is increased in the hippocampus of the LPS‐induced mouse model and 5xFAD mouse model, further supporting its relationship with inflammation‐based cognitive dysfunction. Mechanistically, FMN regulates RFK via epigenetic regulation of KMT2B, as KMT2B overexpression conversely abolishes the effects of FMN on inflammation. Because RFK expression is dependent on the AD pathology,^[^ ^10^ ^]^ FMN may provide an alternative intervention approach for cognitive dysfunction via activation of the riboflavin pathway. While FAD showed little therapeutic efficacy in our experimental system, its activity has been associated with metabolic diseases, such as multiple acyl‐coenzyme A dehydrogenase deficiency and hepatic steatosis.^[^ ^18^ ^]^ On the other hand, FMN has additional roles in generating ATP and NADH, which are novel regulators of calcium homeostasis, mitochondrial functions, and aging.^[^ ^19^ ^]^ Therefore, additional research is needed to clarify the distinct roles of FMN cofactors in neurodegenerative diseases. Estrogen plays a vital role in regulating memory,^[^ ^20^ ^]^ and it also could modulate riboflavin pathways (such as riboflavin carrier protein).^[^ ^21^ ^]^ In this study, we wanted to preclude the effects of estrogen, so we performed the studies only in male mice.

Though microglia are key players in inflammation‐related cognitive dysfunction, they are resistant to manipulation by recombinant viruses such as lentiviruses and adeno‐associated viruses.^[^ ^22^ ^]^ Furthermore, the BBB deters pharmacological treatments. Consequently, a variety of engineered technologies have emerged for neurodegenerative and neurological diseases, such as the photoresponsive vaccine‐like chimeric antigen receptor and stem cell‐derived extracellular vesicle systems designed to targeting microglia in inflammation‐related depression and cerebral ischemia.^[^ ^23^ ^]^ In this study, we establish a biomimetic microglial nanoparticle system (MNPs@FMN) to specifically deliver FMN to microglia. In vitro, ex vivo, and in vivo models confirmed the efficacy of delivering MNP to microglia using this approach. Intriguingly, the in vivo results are suggestive of slow release of FMN from the MNPs@FMN, providing a potential of long‐lasting and controlled release of this system. Our results demonstrate that MNPs@FMN significantly ameliorates cognitive deficits and dysfunctional synaptic plasticity and attenuates inflammatory response in the LPS‐induced mouse model and 5xFAD mouse model of dementia. As the most abundant plasma protein in humans, HSA is natural and stable. Furthermore, HSA is a non‐toxic, non‐immunogenic endogenous protein with high biocompatibility, biodegradability, and good biosafety, so it provides an ideal carrier material. At present, HSA nanoparticles, such as Abraxane, have been approved by the US Food and Drug Administration (FDA) for clinical treatment.^[^ ^24^ ^]^ Thus, we chose HSA as a carrier to target the BBB that is conducive to future clinical use. Astroglial activation is also a vital cause of neuroinflammation,^[^ ^25^ ^]^ and although we did not observe co‐localization between RFK and astrocytes, our results suggest that microglial FMN supplementation suppresses astroglial activation, indicating that it may affect the glial interplay, which needs further study.

In this study, we identified three candidate genes (*Uqcrb*, *Cox8b*, and *Calml4*) that may be related to FMN's neuroprotection. Uqcrb is a subunit of mitochondrial complex III in the mitochondrial respiratory chain,^[^ ^26^ ^]^ and its expression is reported to be correlated with colorectal cancer and glioblastoma.^[^ ^27^ ^]^ Furthermore, Uqcrb inhibits hypoxia‐induced mitochondrial ROS generation in tumor cells and displays anti‐angiogenic activity.^[^ ^28^ ^]^ As an OXPHOS subunit, mitochondrial Cox8b is critical for cellular respiration and cytochrome c oxidase activity,^[^ ^29^ ^]^ with additional functionality in the regulation of thermogenic capacity in adipocyte tissue.^[^ ^30^ ^]^ The EF‐hand protein Calml4 functions as a light chain for Myo7b, is an adhesion complex component, and may be related to hereditary deaf‐blindness.^[^ ^31^ ^]^ In the inflammation‐based mouse model, FMN‐reversed LPS‐reduced *Uqcrb*, *Cox8b*, and *Calml4* mRNA expression, and RFK overexpression abolished FMN's effects, suggesting that the expression of these genes may be linked with the protective effects of FMN on mitochondrial function. Therefore, our findings offer insights for FMN function as a therapeutic intervention for inflammation‐based cognitive decline.

## 4 Experimental Section

### Materials

FMN (GC44836) and FAD (GC11941) were purchased from GlpBio Technology (Montclair, California, USA). Anti‐TNFR1 (H‐5, sc‐8436) and RFK (E‐7, sc‐398830) antibodies were purchased from Santa Cruz Biotechnology (Dallas, TX, USA). Anti‐Phospho‐NF‐*κ*B p65 (#3033), NF‐*κ*B p65 (#8242), and KMT2B (#47 097) antibodies were purchased from Cell Signaling Technology (Danvers, MA, USA). Anti‐Iba1 (019–19741) antibody was purchased from FUJIFILM Wako (Osaka, Japan). Anti‐RFK (15813‐1‐AP) and GAPDH (60004‐1) antibodies were purchased from the Proteintech Group (Rosemont, IL, USA). Anti‐Phospho‐I*κ*B*α* (ab133462), Iba1 (ab178847), and I*κ*B*α* (ab32518) antibodies were purchased from Abcam (Cambridge, MA, USA). Anti‐GFAP (MAB360) and NeuN (MAB377) antibodies were purchased from Millipore (Billerica, MA, USA). Purified anti‐*β*‐Amyloid, 1–16 antibody (6E10) was purchased from BioLegend (San Diego, CA, USA). DyLight 488 goat anti‐mouse IgG (H+L) (70‐GAM4882) and DyLight 594 goat anti‐rabbit IgG (H+L) (70‐GAR5942) antibodies were purchased from Multi Sciences (Hangzhou, China). Horseradish peroxidase (HRP)‐labeled goat anti‐rabbit IgG and HRP‐labeled goat anti‐mouse IgG were purchased from Beyotime Biotechnology (Shanghai, China). Human serum albumin was obtained from Shanghai Yuanye Bio‐Technology Co., Ltd (Shanghai, China). Fluorescent Cy5.5 dye was purchased from ThermoFisher Scientific (Waltham, MA, USA).

### Cell Culture and Drug Treatment

BV2 cells were purchased from American Type Culture Collection (ATCC, Manassas, VA, USA) and were cultured in RPMI‐1640 supplemented with 10% fetal calf serum (FBS) (GIBCO, Carlsbad, CA, USA) at 37 °C and 5% CO_2_. Primary microglia were obtained according to our previous study,^[^ ^32^ ^]^ and they were cultured in Dulbecco's modified Eagle's medium/F12 (DMEM/F12, GIBCO, Carlsbad, CA, USA) supplemented with 10% FBS and GM‐CSF at 37 °C in 5% CO_2_.

BV2 cells and microglia were plated onto 6‐well plates at 5 ×  10^5^ cells and 3 ×  10^5^ cells per well, respectively. Stock solutions of LPS (2.5 mg mL^−1^), FMN (50 mg mL^−1^), and FAD (50 mg mL^−1^) were prepared in 0.01 M PBS and diluted in cell culture medium to the appropriate working concentrations. LPS (1 µg mL^−1^) was used to treat cells for 6 h to induce inflammation. The dose of LPS used in this study was selected according to the study of Nam et al.^[^ ^33^ ^]^ For detecting the anti‐inflammatory effects of FMN and FAD, the medium was removed after 6 h LPS treatment, and the cells were then treated with different concentrations of FMN and FAD (5, 50, 100 and 200 µM) for another 24 h. The doses and incubation times of FMN and FAD were selected according to our previous study.^[^ ^17^ ^]^

### Rfk and Kmt2b SiRNA Construction

SiRNA targeting *Rfk* sequences (siRNA‐1, 5′‐GGACAATCTTCCAGCTGAT‐3′; siRNA‐2, 5′‐CACTTATTTCTGCAATTCA‐3′; siRNA‐3, 5′‐CCAAGTTTCTAAAGGCAAA‐3′), and *Kmt2b* sequences (siRNA‐1, 5′‐ GCCCTACCACTCACTATGT‐3′; siRNA‐2, 5′‐ GCAGAATGAGTGGACACAT‐3′; siRNA‐3, 5′‐ GCATCAACTTCAAGCGAAA‐3′) were designed and synthesized by RiboBio Co., Ltd (Guangzhou, China), and negative‐control siRNA was also provided by RiboBio Co., Ltd. SiRNA transfection was performed according to our previous study using Lipo8000 Transfection Reagent (Beyotime Biotechnology).^[^ ^34^ ^]^ To investigate the anti‐inflammatory effects of *Rfk* and *Kmt2b* knockdown, the cells were transfected with siRNA for 72 h, and then LPS was added for 6 h.

### Extraction of Microglial BV2 Cell Membrane

BV2 cell membranes were obtained to assemble BMNP according to the previously reported extrusion approach.^[^ ^35^ ^]^ After applying RIPA lysis buffer and mechanical membrane disruption (VCX130 ultrasonics processor, USA) for 20 s, differential centrifugation was performed (Thermo Fisher Scientific, Waltham, MA, USA) to eliminate the intracellular contents of BV2 cells, and the BV2 cell membranes were obtained.

### Formulation of MNPs@FMN

FMN and HSA were dissolved and mixed in deionized water, and then CHCl_3_ was added to the mixture. After sonication using an ultrasonics processor at 30 W and 20 kHz for 5 min, the nanoparticle core (NPs@FMN) was prepared. Then, the extracted BV2 cell membranes and 100 µL of DSPE‐PEG2000 (10 mg mL^−1^) lipid dispersion were combined with the NPs@FMN. The mixture was extruded through a polycarbonate membrane for ≈10 passes to form MNPs@FMN. Ultimately, the obtained nanoparticles were dialyzed with PBS (pH 7.4) for 24 h at room temperature.

### Characterization of the MNPs@FMN

The size distribution, polydispersity index, and *ζ*‐potential of NPs@FMN and MNPs@FMN were investigated using the Zetasizer Nano ZS (Malvern, U.K.). The morphology of NPs@FMN and MNPs@FMN was observed by transmission electron microscopy (FEI Tecnai G2 F20 S‐Twin, USA). Absorption spectra were obtained using UV–vis spectrometry (Lambda 35, Perkin‐Elmer, USA).

### Protein Profile Analysis of MNPs@FMN

The protein concentrations of BV2 cell lysates, extracted BV2 cell membranes, and prepared MNPs@FMN were determined using a BCA assay kit (Beyotime, Shanghai, China). Then, the samples were subjected to SDS‐PAGE using the Mini‐PROTRAN Tetra System (BIO‐RAD, CA, USA). Immunoblots were obtained using the GeneGnome XRQ Chemiluminescence imaging system (Gene Company, Hong Kong, China).

### Drug Release Profile of MNPs@FMN

The release kinetics of FMN under mimic physiological conditions from NPs@FMN and MNPs@FMN were measured by a dialysis method. In brief, 1 mL NPs@FMN or MNPs@FMN (containing 1 mg mL^−1^ FMN) was suspended in 10 mL of PBS (pH 7.4) in a dialysis bag with a molecular weight cutoff of 100 kDa. The solution was dialyzed against 30 mL of PBS (pH 7.4) under 200 rpm stirring at 37 °C. At different time points, the absorbance of the resulting FMN was measured by UV–vis spectrometry, and the drug release kinetics were determined using a standard curve of FMN in PBS with absorption at 450 nm.

### Encapsulation Efficiency and Drug Loading

The encapsulation efficiency of NPs@FMN and MNPs@FMN was determined by ultrafiltration. Briefly, free FMN and NPs@FMN or MNPs@FMN were separated by ultracentrifugation (Millipore, Billerica, MA, USA). NPs@FMN or MNPs@FMN was retained, and free FMN was separated from the filtrated through the ultracentrifugation. The initial concentration of FMN in NPs@FMN or MNPs@FMN and free FMN were determined by UV–vis spectrometry. The encapsulation efficiency was calculated as follows:

(1) $$\mathrm{EE}\%=\left(Wt-Wf\right)/Wt\times 100\%.$$

where *W*_t_ refers to the initial concentration of the FMN to the NPs@FMN or MNPs@FMN, and *W*_f_ refers to the free FMN in the filtrate.

The collected samples of NPs@FMN and MNPs@FMN were lyophilized, and 1 mg freeze‐dried powder of NPs@FMN and MNPs@FMN were dissolved thoroughly in ddH_2_O and analyzed by UV–vis spectrometry. The loading efficiency was calculated as follows:

(2) $$\mathrm{LE}\%=W_{F}/W_{N}\times 100\%.$$

Where *W*_F_ refers to the weight of the FMN in NPs@FMN or MNPs@FMN, and *W*_N_ refers to the weight of NPs@FMN or MNPs@FMN.

### Preparation of MNPs@FMN‐Cy5.5

First, 2 mg FMN, 40 mg HSA and 20 µL Cy5.5 (5 mg mL^−1^) were dissolved in 2 mL deionized water, and then 100 µL CHCl_3_ was added into the mixed solution, which was sonicated for 5 min on an ultrasonics processor at 30 W and 20 kHz to form a nanoparticle core (NPs@FMN‐Cy5.5). Finally, NPs@FMN‐Cy5.5 were coated with BV2 cell membrane to form the MNPs@FMN‐Cy5.5.

### Flow Cytometry

BV2 cells and microglia were seeded at a density of 1 × 10^6^ and 3 × 10^5^ cells per well into 6‐well plates, respectively. After 24 h incubation, the culture medium was replaced with fresh medium containing NPs@FMN or MNPs@FMN (10 µg mL^−1^ of FMN, 0.5 µg mL^−1^ of Cy5.5). After 3 h incubation, the cells were washed with PBS and collected for flow cytometry analysis (Ex: 638 nm, Em: 712/25 nm) (Beckman Cytoflex, USA).

### In Vitro Evaluation of BBB Permeability of MNPs@FMN

In vitro BBB permeability evaluation was performed according to the methods of a previous study.^[^ ^36^ ^]^ Approximately 1 × 10^5^ – 1 × 10^6^ bEnd.3 cells were seeded in polyester transwell inserts (6 wells, pore diameter of 0.4 µm, 4.67 cm^2^, Corning, NY, USA), and a Millicell‐ERS volt‐ohmmeter (Millipore, Billerica, MA, USA) was used to detect the Trans‐Epithelial Electrical Resistance (TEER) of the bEnd.3‐cell monolayer to confirm the tightness of the monolayer to simulate the BBB. To evaluate the BBB permeability of MNPs@FMN across the monolayer, the culture media on the apical side was replaced with PBS, NPs@FMN or MNPs@FMN (0.5 µg/mL of Cy5.5 in 0.7 mL) for 3 h, and the lower side culture media was replaced with fresh DMEM. The fluorescence of the insert of the transwell and the bottom chamber of the bEnd.3 layer was imaged using a confocal laser‐scanning microscope (SP8; Leica, Hamburg, Germany).

### Ex Vivo Imaging and Biodistribution Analysis

Ex vivo evaluation of MNPs@FMN brain delivery was conducted as previously described.^[^ ^37^ ^]^ Briefly, mice were intravenously injected with NPs@FMN or MNPs@FMN (200 µL, containing 1 mg mL^−1^ FMN and 50 µg mL^−1^ Cy5.5). The real time (0.5, 1, 3, 6, 9,12, and 24 h) fluorescence was quantitatively analyzed using the IVIS imaging system (Excitation wavelength: 674 nm, Emission wavelength: 692 nm). After 24 h, the major organs (heart, liver, spleen, lung, kidneys, brain) were sacrificed and analyzed using the IVIS imaging system.

### Animals and Drug Treatments

Adult (8‐week‐old) male C57BL/6J mice were purchased from SPF Biotechnology Co., Ltd. (Beijing, China). Male 5xFAD mice (10‐month‐old) were purchased from the Jackson Laboratory (Bar Harbor, ME, USA). The age‐ and sex‐matched wildtype (WT) mice were used as control. The mice were housed in groups of four under a 12/12 h light/dark cycle with ad libitum access to food and water. The animal experimental protocols were in compliance with the Institutional Animal Care and Use Committee of Guangzhou Medical University (Approval number: GY2020‐041) and National Institute of Health guidelines on the care and use of animals (NIH Publications No. 8023, revised 1978).

To examine the effects of FMN and FAD on RFK expression, different doses of FMN and FAD (0, 5, 10, 20, 40 mg/kg) were intraperitoneally injected three times a week for 5 weeks. The mice were sacrificed, and hippocampal samples were collected and analyzed. The control group was injected with PBS.

To compare the effects of *Rfk* knockdown with free FMN on inflammation‐based cognitive dysfunction, mice were injected with lentivirus (LV)‐packaged Ctrl or Rfk shRNA for 3 weeks. Afterward, LPS (0.25 mg kg^−1^) was administered intraperitoneally for 7 consecutive days. Then, LV‐Ctrl mice were intraperitoneally injected with PBS or FMN (40 mg kg^−1^) three times a week for 5 weeks. LV‐Rfk mice were intraperitoneally injected with PBS.

To investigate the effects of MNPs@FMN on inflammation‐based cognitive dysfunction, LPS (0.25 mg kg^−1^) was administered intraperitoneally for 7 consecutive days, and simultaneously, NPs@FMN, MNPs@FMN (equivalent dose of FMN), or unloaded MNPs vehicle were administered intravenously starting on the day of the first LPS injection once every other day for 11 days. After 3 days, behavioral tests were performed.

To study the effects of MNPs@FMN on the AD mouse model, 5xFAD mice were given intravenously free FMN or MNPs@FMN every other day for 11 days. The control group was given intravenously PBS. After 3 days, behavioral tests were performed.

### Stereotaxic Injection of LV‐Rfk shRNA in the Hippocampus

LV‐Ctrl and LV‐Rfk were packaged by Sunbio Medical Biotechnology (Shanghai, China) and they were stereotaxically injected in the hippocampus according to our recent work.^[^ ^17^ ^]^ Briefly, mice were anesthetized and placed in a stereotaxic frame. LV‐Ctrl and LV‐Rfk in 0.5 µL vol were delivered into the bilateral hippocampus at the target site (Bregma AP, −2.0 mm, ML, ±2.0 mm, DV, −2.0 mm). The syringe was left in place for 5 min before being slowly withdrawn from the brain.

### Open Field Test

The OFT was performed according to the previous report.^[^ ^38^ ^]^ In this study, mice were placed in a rectangular plastic box (40 × 40 × 40 cm). The movement was recorded for 15 min using a video tracking system (EthoVisione XT software, Beijing, China). The total distance, movement speed, number of entries to the center zone, and time spent in the central zone were analyzed.

### Y‐Maze Test

The Y‐maze test was used to evaluate spontaneous alternations as a measure of the spatial working memory, according to previous methods.^[^ ^39^ ^]^ The Y maze apparatus was constructed from gray plastic and consisted of three arms (30 cm long, 10 cm wide, and 20 cm high). Mice were placed within the center zone and were allowed to explore the Y‐maze freely for 8 min. The time spent in each arm was calculated visually (EthoVisione XT software, Beijing, China). Non‐overlapping entrance sequences were defined as spontaneous alternations (%).

### Morris Water Maze (MWM)

The MWM test was performed as previously described.^[^ ^40^ ^]^ The test apparatus consisted of a circular pool with a diameter of 120  cm and was filled with water (22 ± 1 °C) made opaque white with bright white food coloring. The circular pool was divided into four quadrants, and different images (circles, squares, and triangles) were hung on the pool walls. During the 5‐day training, the mice were placed in the water randomly in one of the four quadrants to look for the hidden platform for 60 s, and they were allowed to stay on the platform for 20 s. If the mice failed to find the platform, they were guided towards it, where they remained for 20 s. The time to find the platform was recorded by a computerized tracking system (EthoVision XT; Beijing, China). On the sixth day of the probe test, the platform was removed, and the mice were allowed to swim freely for 60 s. The moving track and time spent in the target quadrant were recorded using a video tracking system (EthoVisione XT software, Beijing, China).

### Quantitative Reverse Transcription Polymerase Chain Reaction (qRT‐PCR)

Cultured cells or hippocampal samples were harvested, and total RNA was isolated using Trizol reagent (Invitrogen, San Diego, CA, USA). The RNA was used to generate cDNA using a cDNA Reverse Transcription Kit (QIAGEN, Waltham, MA, USA). Realtime PCR was carried out with TB Green Premix Ex Taq (Takara, Shiga, Japan). Results were obtained using the 2^−ΔΔ^ *^CT^* method as described previously.^[^ ^41^ ^]^ Data were obtained from three separate experiments, each of which was performed in triplicate. Gene expression was normalized to GAPDH. Primer sequences are listed in Table S2, Supporting Information.

### Western Blotting Assay

Total protein was extracted from cultured cells or hippocampal samples using RIPA Lysis Buffer (Beyotime Biotechnology, Shanghai, China). The total protein (30–40 µg) was separated on 6%–12% gel by SDS‐PAGE and electrophoretically transferred onto polyvinylidene difluoride membranes. After blocking, the membranes were incubated with primary antibodies, washed, and incubated with HRP‐conjugated secondary antibodies. The immunoblots were visualized using the GeneGnome XRQ Chemiluminescence imaging system (Gene Company, Hong Kong, China). Image J software was used to analyze the optical density of the bands.

### Enzyme‐linked Immunosorbent Assay (ELISA)

ELISA was performed as previously described.^[^ ^37^ ^]^ In brief, hippocampal tissues were homogenized in PBS buffer and centrifuged at 12 000 × *g* for 20 min at 4 °C, and then the supernatants were collected. The concentrations of IL‐1*β*, IL‐6, and TNF‐*α* in the hippocampus and cultured cellular supernatants were measured using ELISA kits (Enzyme‐linked Biotechnology Co., Ltd., Shanghai, China) according to the manufacturer's instructions. OD values were measured by Multiscan Spectrum (PerkinElmer, MA, USA) at 450 nm, and the results were expressed as pg per mg protein (pg mg^−1^ protein) or pg mL^−1^.

### Immunofluorescence Assay

Mouse brains were fixed in 4% paraformaldehyde and dehydrated with 20%–30% sucrose buffer. The fixed brains were then sectioned using a freezing microtome (Leica, Hamburg, Germany). Afterwards, the brain sections or cultured cells were blocked with 5% BSA, and incubated with primary antibodies and then with fluorescent‐labeled secondary antibodies. Images were captured using a confocal laser‐scanning microscope (SP8; Leica, Hamburg, Germany). Quantitative analysis was performed using the Image‐Pro Plus 6.0 photogram analysis system (IPP 6.0, Media Cybernetics, Bethesda, MD, USA).

### RNA‐seq and Bioinformatic Analysis

Total RNA was isolated from hippocampal samples using Trizol reagent (Invitrogen, San Diego, CA, USA), and the Agilent 2100 bioanalyzer (Agilent Technologies, CA, USA) was used to examine the RNA integrity. RNA libraries were prepared using a NEBNext Ultra RNA Library Prep Kit for Illumina (New England Biolabs [NEB], Ipswich, MA, USA) according to the manufacturer's instructions, and index codes were added to attribute sequences to each sample. All downstream analyses were performed with high‐quality clean data, and sequencing was performed on an Illumina HiSeq 2500 platform (San Diego, CA). Statistically significant DEG analysis was performed using the DESeq2 R package (1.10.1). The resulting *p*‐values were adjusted using the Benjamini‐Hochberg approach to control for false discovery rates. DEGs were defined by an adjusted *p*‐value < 0.05 and absolute values of Log_2_ (fold change) > 1. The DEGs were subjected to GO and KEGG enrichment analyses.

### Electrophysiology

Following behavioral analysis, ex vivo hippocampal slices were prepared from mice injected with either LPS or FMN‐encapsulated nanoparticles using methods described previously.^[^ ^41^ ^]^ Briefly, mice were decapitated under deep terminal anesthesia, and their brains were surgically removed in ice‐cold, sucrose‐based artificial cerebrospinal fluid (aCSF) (64 mM NaCl, 2.5 mM KCl, 1.25 M NaH_2_PO_4_, 10 mM MgSO_4_, 0.5 mM CaCl_2_, 26 mM NaHCO_3_, 10 mM glucose, and 120 mM sucrose) saturated with carbogen (95% O_2_/5% CO_2_) at pH 7.3–7.4. A vibrating‐blade microtome (Leica VT1200S) was used to cut coronal slices of hippocampus 400 µm in thickness. Slices were transferred to a holding chamber containing warm (34 °C) recording aCSF (126 mM NaCl, 2.5 mM KCl, 1.25 mM NaH_2_PO_4_, 2 mM MgSO_4_, 2 mM CaCl_2_, 26 mM NaHCO_3_, and 10 mM glucose) saturated with carbogen (95% O_2_/5% CO_2_) at pH 7.3–7.4. Slices were left to recover for 30 min at 34 °C and then at room temperature for at least 30 min before recording. The fEPSPs were evoked in the CA1 stratum radiatum by stimulating the SCs pathway with a concentric bipolar stimulating electrode (CBARC75; FHC), input–output relations were applied in increments of 50 µA from 0 to 500 µA, LTP was induced by two trains of high‐frequency stimulation (100 Hz for 1 s at 30‐s intervals), fEPSPs were recorded using aCSF‐filled glass pipettes (3–5 MΩ). All recordings of synaptic activity were performed using a Multiclamp 700B (Molecular Devices, San Jose, CA, USA), and sampled using pClamp11 software (Molecular Devices, San Jose, CA, USA).

### UPLC‐MS/MS Analysis

FMN concentrations in the hippocampus after intraperitoneal delivery of free FMN, and intravenous delivery of PBS, free FMN, or MNPs@FMN were examined using UPLC‐MS/MS analysis. Briefly, each tissue sample (≈15 mg) was mixed with 20 µL of deionized water and homogenized for 3 min, and 150 µL of methanol containing internal standard was added to extract the metabolites. The sample was homogenized for another 3 min and then centrifuged at 18 000 × *g* for 20 min (Microfuge 20R, Beckman Coulter, Inc., Indiannapolis, IN, USA). Then the supernatant was transferred to a 96‐well plate and subjected to a UPLC‐MS/MS system (Acquity UPLC with Xevo TQ‐S Mass Spectrometer, Waters Corp., Milford, MA, USA). Samples were injected onto an Atlantis Premier analytical column (100 × 2.1 mm, 1.7 µm) at a flow rate of 0.4 mL min^−1^. The Q Exactive series mass spectrometer was operated in positive/negative polarity mode with a spray voltage of 3 kV. The raw data files generated by the UPLC‐MS/MS were processed using MassLynx software (v4.1, Waters, Milford, MA, USA) to perform peak alignment, peak picking, and quantitation for FMN.

### Liver and Kidney Function Analysis

Serum was collected after the mice were sacrificed and was centrifugated at 3000 × *g* for 10 min. Then, the serum was subjected to liver and kidney function analysis using the Hitachi 7180 automatic biochemical analyzer (Tokyo, Japan). The rate method was used to measure aspartate aminotransferase (AST), alanine aminotransferase (ALT), alkaline phosphatase (ALP), and lactate dehydrogenase (LDH); the hexokinase method was used to measure glucose (GLU); the enzymatic method was used to measure creatinine (Cr) and UREA; the uricase ultraviolet method was used to measure uric acid (UA); the bromocresol green method was used to measure albumin (ALB); the biuret method was used to measure total protein (TP) and globulin (GLOB).

### Statistical Analysis

Gaussian distribution of the results was checked by Shapiro ‐Wilk test and *p* ≥ 0.05 using SPSS software (version 17.0, SPSS Inc. Chicago, IL, USA). Statistical analysis was performed using Prism 9.0 (GraphPad Software, La Jolla CA). One‐way analysis of variance (ANOVA) followed by the Tukey's *post‐hoc* test was used for multiple comparisons, whereas the Student's *t*‐test was used for comparisons between two groups. All calculations for sample sizes were done using an online sample size calculator (https://clincalc.com/stats/samplesize.aspx), and sample sizes were chosen based on the means and variation of preliminary data to achieve at least 80% power and allow for a 5% type I error. Data are presented as mean ± standard error of the mean (SEM), and the statistical significance level was set at *p* < 0.05.

## Conflict of Interest

The authors declare no conflict of interest.

## Supporting information

Supporting Information

## Data Availability

The data that support the findings of this study are available from the corresponding author upon reasonable request.
