# Supplementary material for: Biomimetic Remodeling of Microglial Riboflavin Metabolism Ameliorates Cognitive Impairment by Modulating Neuroinflammation
Source: Adv Sci (Weinh). 2023 Feb 17;10(12):2300180. doi: 10.1002/advs.202300180 (PMC10131853; doi:10.1002/advs.202300180)
Supplement: Supplementary file 1 — Supporting Information [file ADVS-10-2300180-s001.pdf]

ADVANCED SCIENCE
Open Access

Supporting Information

for *Adv. Sci.*, DOI 10.1002/advs.202300180

Biomimetic Remodeling of Microglial Riboflavin Metabolism Ameliorates Cognitive Impairment by Modulating Neuroinflammation

*Mengran Zhang, Huaqing Chen, Wenlong Zhang, Yan Liu, Liuyan Ding, Junwei Gong, Runfang Ma, Shaohui Zheng and Yunlong Zhang**

Supporting Information

Biomimetic Remodeling of Microglial Riboflavin Metabolism Ameliorates Cognitive Impairment by Modulating Neuroinflammation

*Mengran Zhang, Huaqing Chen, Wenlong Zhang, Yan Liu, Liuyan Ding, Junwei Gong, Runfang Ma, Shaohui Zheng, Yunlong Zhang **

M. Zhang, J. Gong, R. Ma, S. Zheng, Prof. Y. Zhang

Department of Neurology, Institute of Neuroscience,

Key Laboratory of Neurogenetics and Channelopathies of Guangdong Province and the Ministry of Education of China,

The Second Affiliated Hospital,

Guangzhou Medical University,

Guangzhou 510260, China.

E-mail: ylzhang@gzhmu.edu.cn

H. Chen

Shenzhen Key Laboratory of Gene and Antibody Therapy, Center for Biotechnology and Biomedicine, State Key Laboratory of Chemical Oncogenomics, State Key Laboratory of Health Sciences and Technology, Institute of Biopharmaceutical and Health Engineering,

Shenzhen International Graduate School,

Tsinghua University,

Shenzhen,

Guangdong 518055, China.

W. Zhang, L. Ding

Department of Neurology,

The First Affiliated Hospital of Guangzhou Medical University,

Guangzhou 510120, China.

Y. Liu

School of Traditional Chinese Medicine,

Jinan University,

Guangzhou 510632, China.

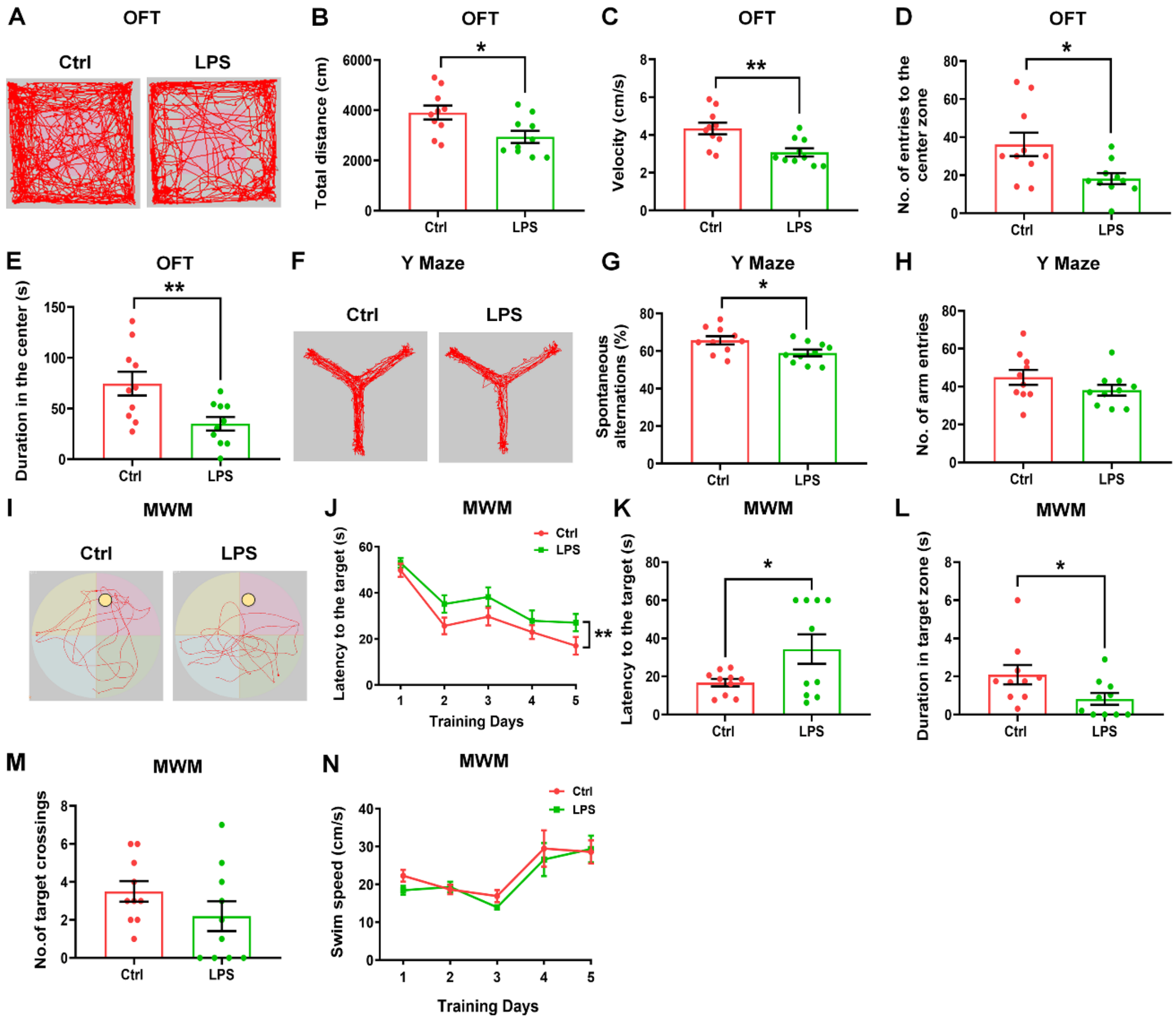

Figure S1. Behavioral assessment of the LPS-induced inflammation-based cognitive dysfunction mouse model. (A-E) Open-field test of the exploratory ability of Control and LPS-treated mice: Travelled tracings (panel A), total distance travelled (panel B), movement speed (panel C), number of entries to the center zone (panel D), and time spent in the center (panel E). (F-H) Y maze test of the working memory of Control and LPS-treated mice: Travelled path tracings (panel F), spontaneous alternations (panel G), and number of arm entries in the Y maze (panel H). (I-N) Morris water maze test of the spatial ability of Control and LPS-treated mice: Representative path tracings in each quadrant during the probe trial (panel I), escape latency during a five-day training course (panel J), average escape latency in the probe test (panel K), time spent in the target zone (panel L); number of target crossings (panel M), swim speed during a five-day training course (panel N). Results are expressed as mean ± SEM. $^{**}p < 0.01$, $^{*}p < 0.05$ vs. Control. Statistical significance was determined using the Student's *t*-test. n = 10 per group.

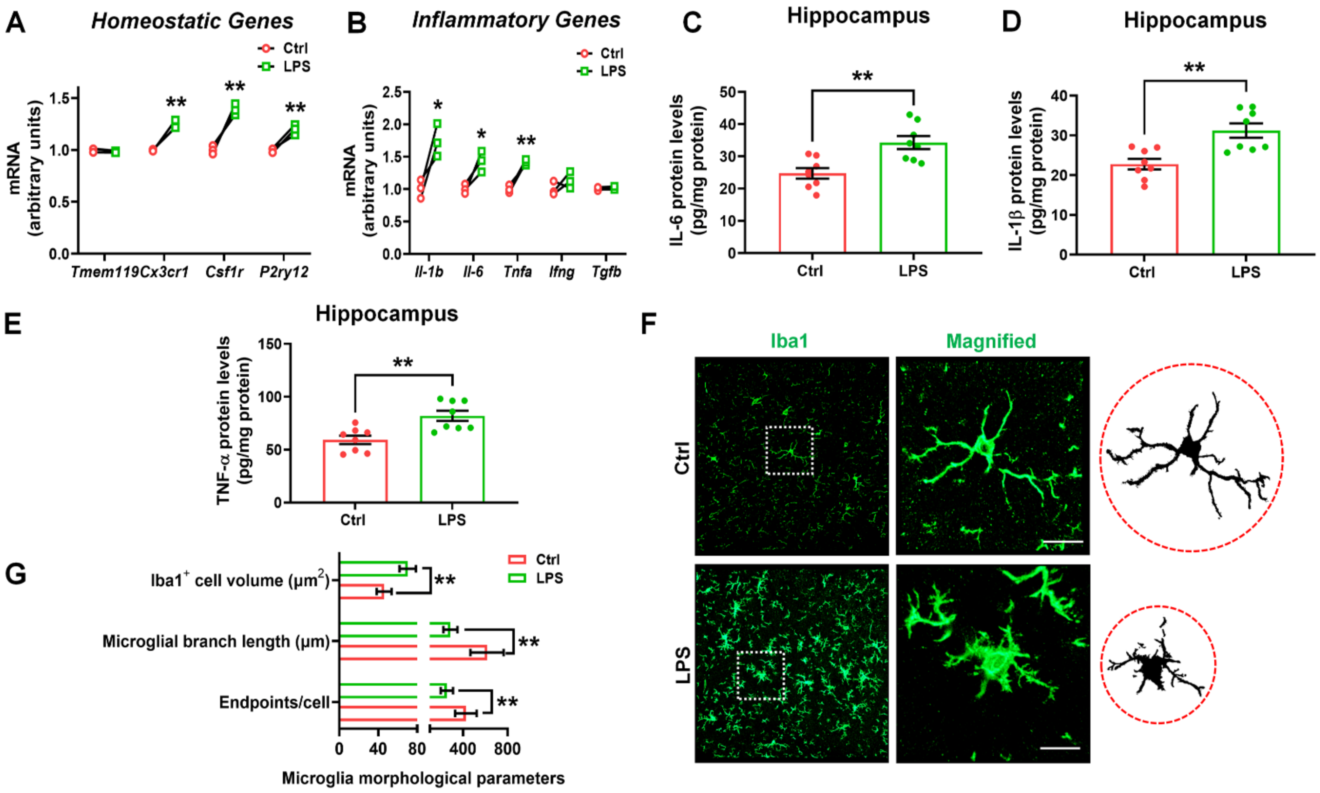

Figure S2. Behavioral tests of LPS-induced mouse model. (A and B) The mRNA expression levels of homeostatic genes (*Tmem119*, *Cx3cr1*, *Csf1r*, *P2ry12*) and inflammatory genes (*Il-1b*, *Il-6*, *Tnfa*, *Ifng*, *Tgfb*) in the hippocampus of Control and LPS mice. n = 3 per group. (C-E) The protein expression levels of IL-1β, IL-6 and TNF-α in the hippocampus of Control and LPS mice. n = 8 per group. (F) Immunofluorescence staining of Iba1-positive cells in the hippocampus of Control and LPS mice. Scale bars, 40 μm. Magnified images and skeletal diagrams of Iba1-positive cells are shown to the right of each staining image. Scale bars, 10 μm. (G) Quantification of the volume, branch length and endpoint voxels of Iba1-positive cells. n = 3 per group. Results are expressed as mean ± SEM. $^{**}p < 0.01$, $^{*}p < 0.05$ vs. Control. Statistical significance was determined using the Student's *t*-test.

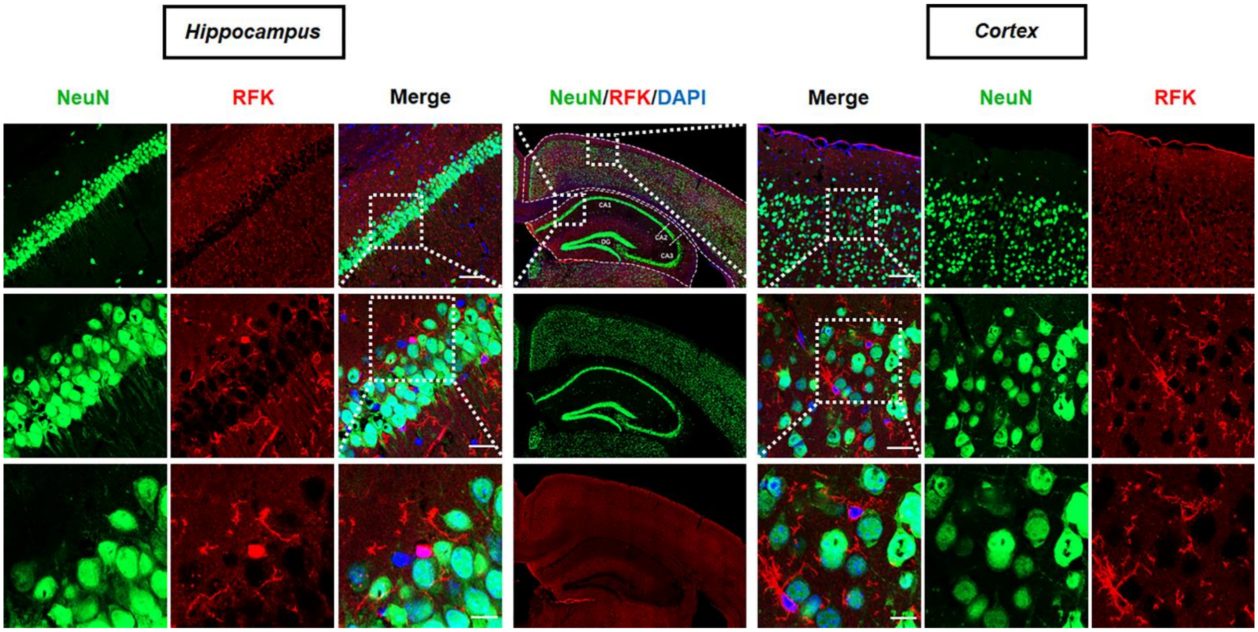

Figure S3. Immunostaining of RFK with NeuN in the hippocampus and cortex. Immunofluorescence staining of RFK with NeuN in the CA1 of hippocampus and cortex. Scale bars, 50 µm (top), 10 µm (middle), and 5 µm (bottom).

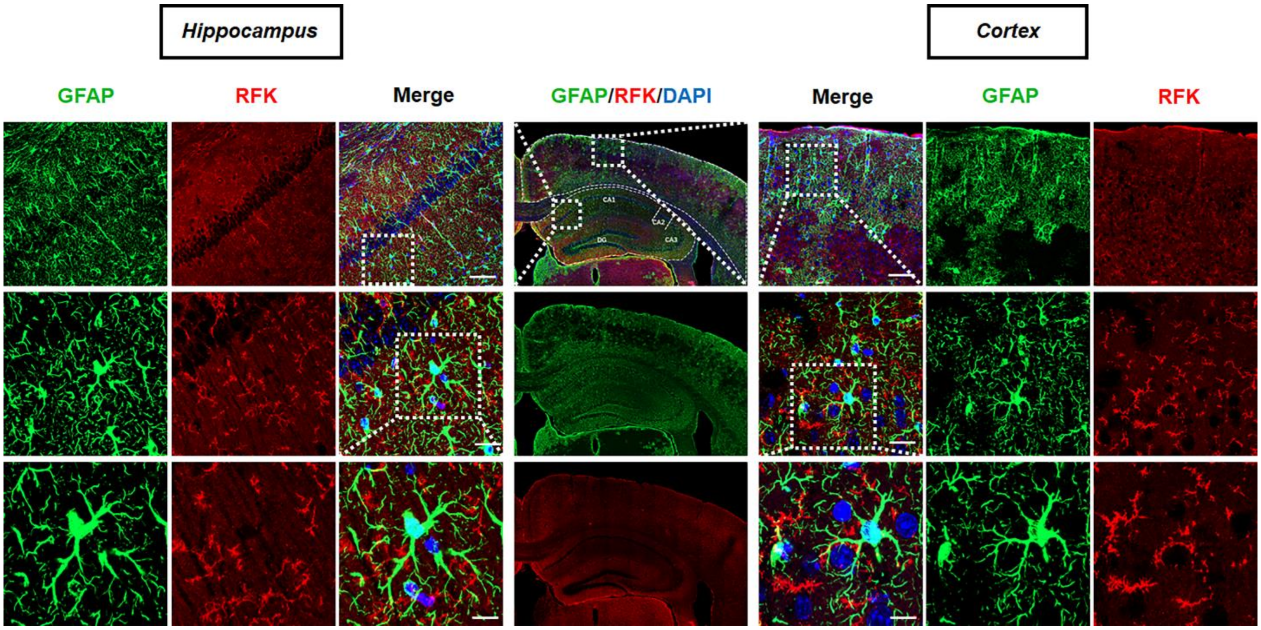

Figure S4. Immunostaining of RFK with GFAP in the hippocampus and cortex. Scale bars, 50 µm (top), 10 µm (middle), and 5 µm (bottom).

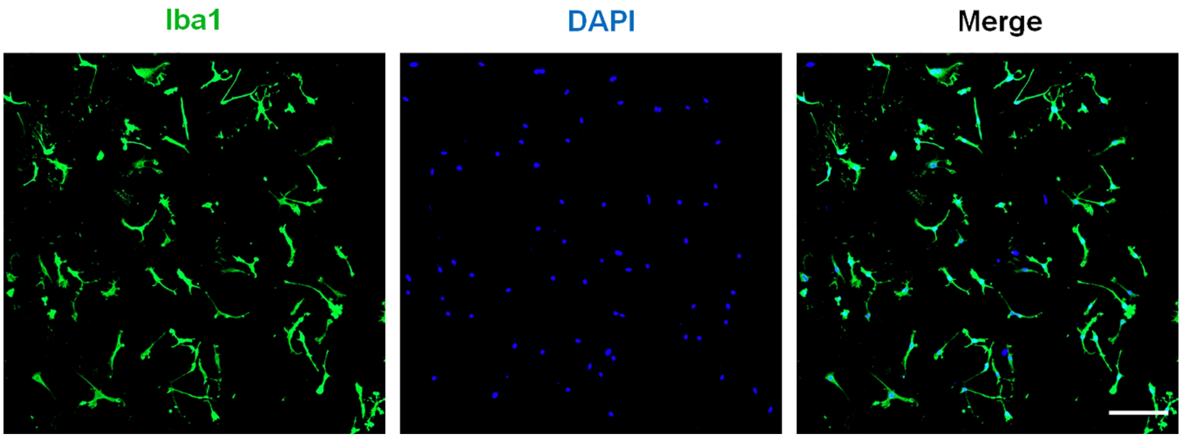

Figure S5. Immunostaining of Iba1 in primary microglia. Immunofluorescence staining of Iba1 in primary microglia. Scale bars, 100 μm.

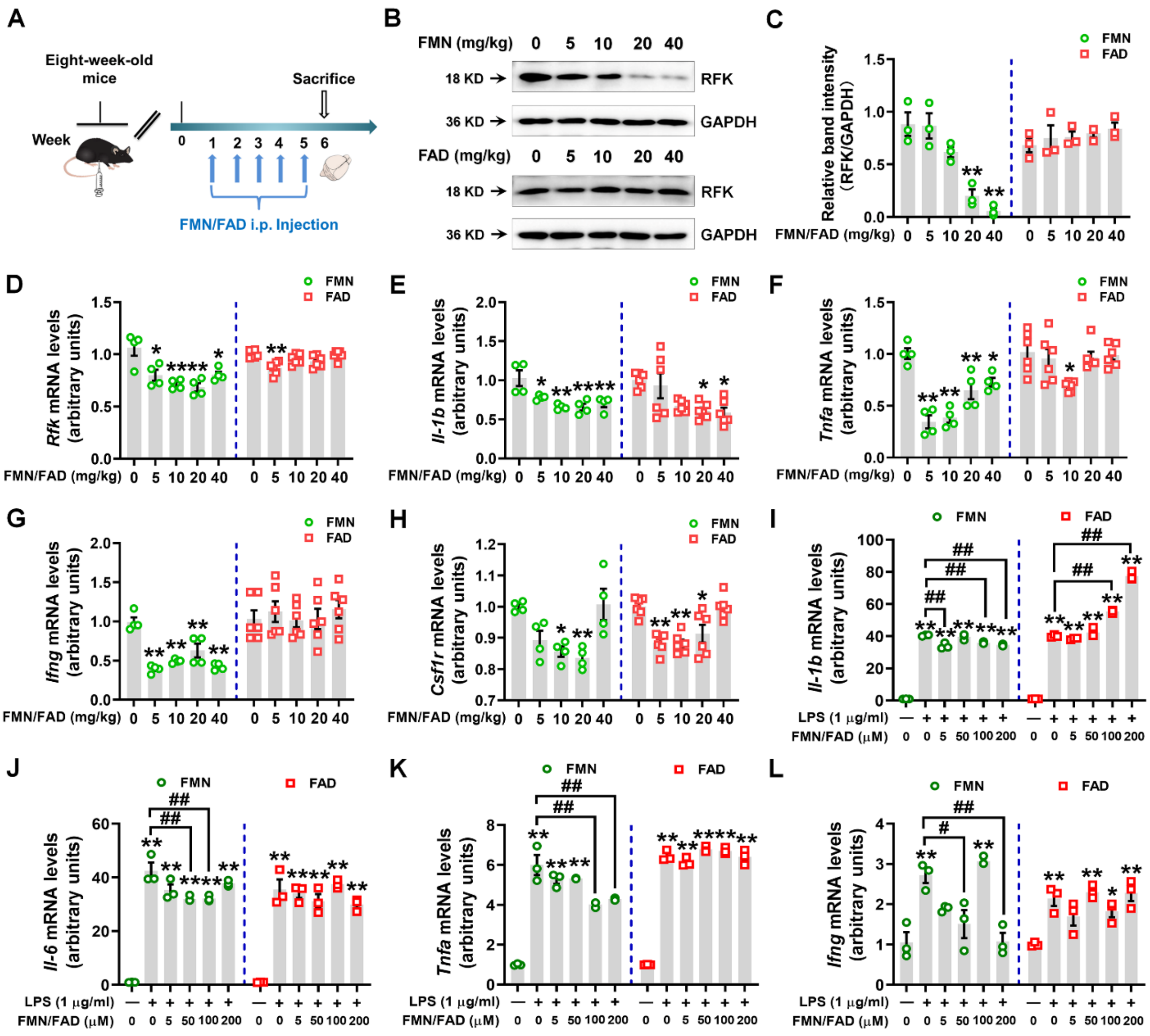

Figure S6. FMN but not FAD decreases RFK expression and pro-inflammatory response. (A) Experimental design for FMN or FAD administration in WT mice. (B and C) Representative blots and quantification showing RFK expression in the hippocampus of WT mice intraperitoneally injected with different doses of FMN or FAD (0, 5, 10, 20, 40 mg/kg). n = 3 per group. (D-H) The mRNA expression levels of *Rfk*, *Il-1b*, *Tnfa*, *Ifng* and *Csf1r* in the hippocampus of WT mice intraperitoneally injected with different doses of FMN or FAD (0, 5, 10, 20, 40 mg/kg). n = 4 per group for FMN and n = 5 per group for FAD. (I-L) The mRNA expression levels of *Il-1b*, *Il-6*, *Tnfa*, and *Ifng* in LPS-treated BV2 cells with different doses of FMN or FAD (0, 5, 50, 100, 200 μM). n = 3 per group. Results are expressed as mean ± SEM. $^{**}p < 0.01$, $^{*}p < 0.05$ vs. Ctrl; $^{\#\#}p < 0.01$, $^{\#}p < 0.05$ vs. LPS. Statistical significance was determined using one-way ANOVA and Tukey's tests for *post hoc* comparisons.

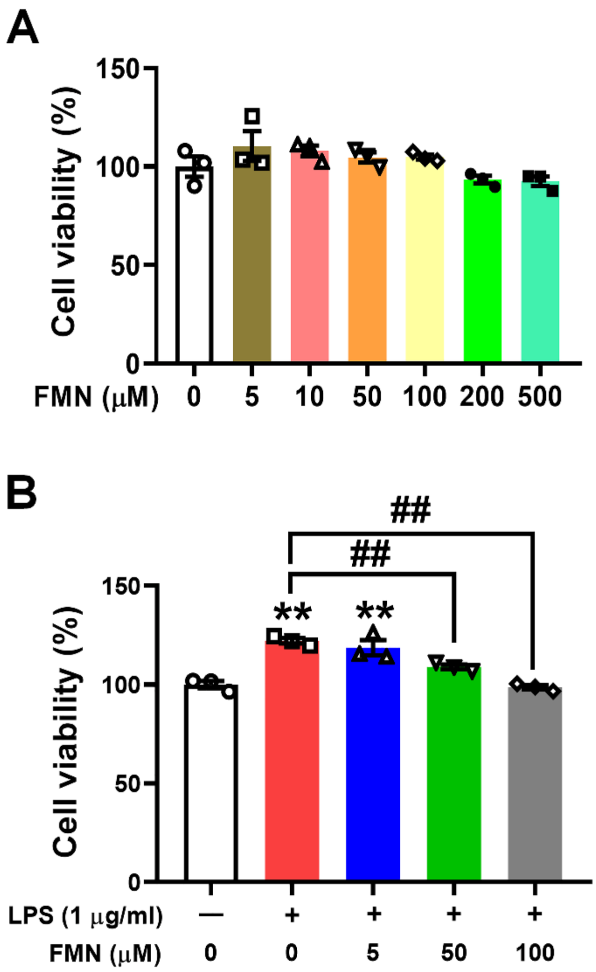

Figure S7. Effects of FMN on the cell viability. (A) Cell viability of BV2 cells treated with different doses of FMN (0, 5, 10, 50, 100, 200 and 500 μM). (B) Cell viability of LPS-treated BV2 cells treated with different doses of FMN (0, 5, 50 and 100 μM). Results are expressed as mean ± SEM. $^{**}p < 0.01$ vs. Control; $^{\#\#}p < 0.01$ vs. LPS. n = 3 per group. Statistical significance was determined using one-way ANOVA and Tukey's tests for *post hoc* comparisons.

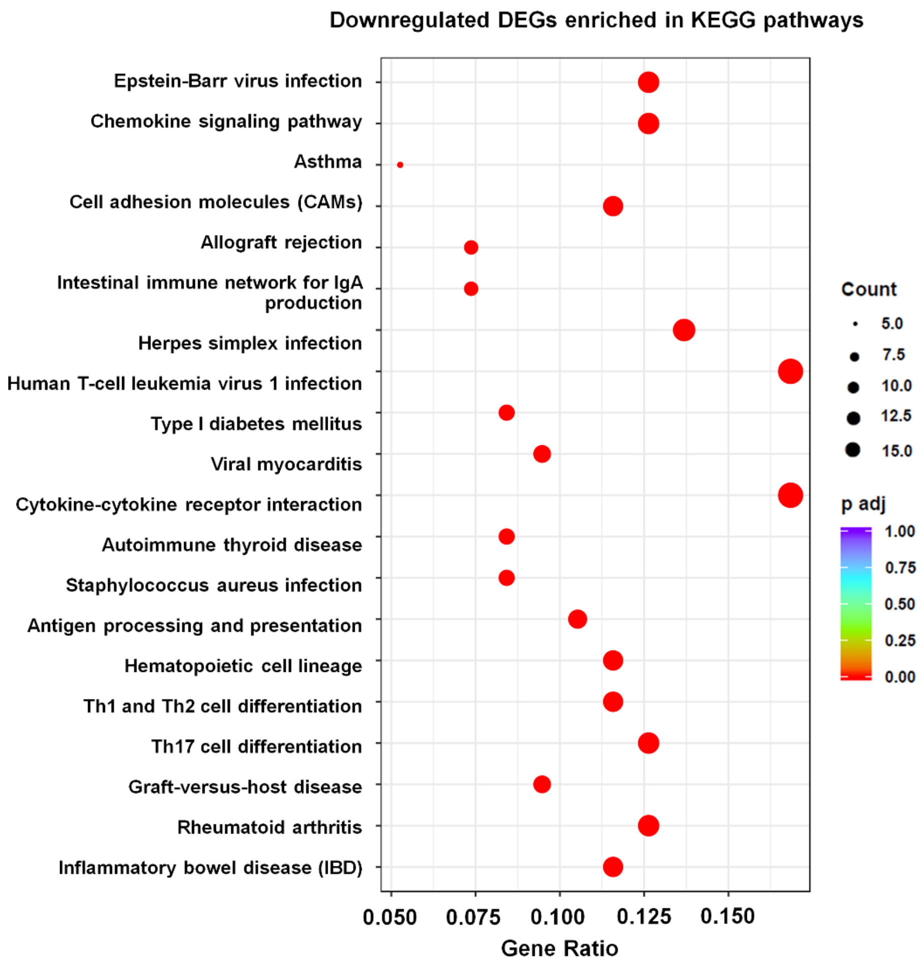

Figure S8. KEGG pathways enriched by downregulated DEGs between FMN and Ctrl.

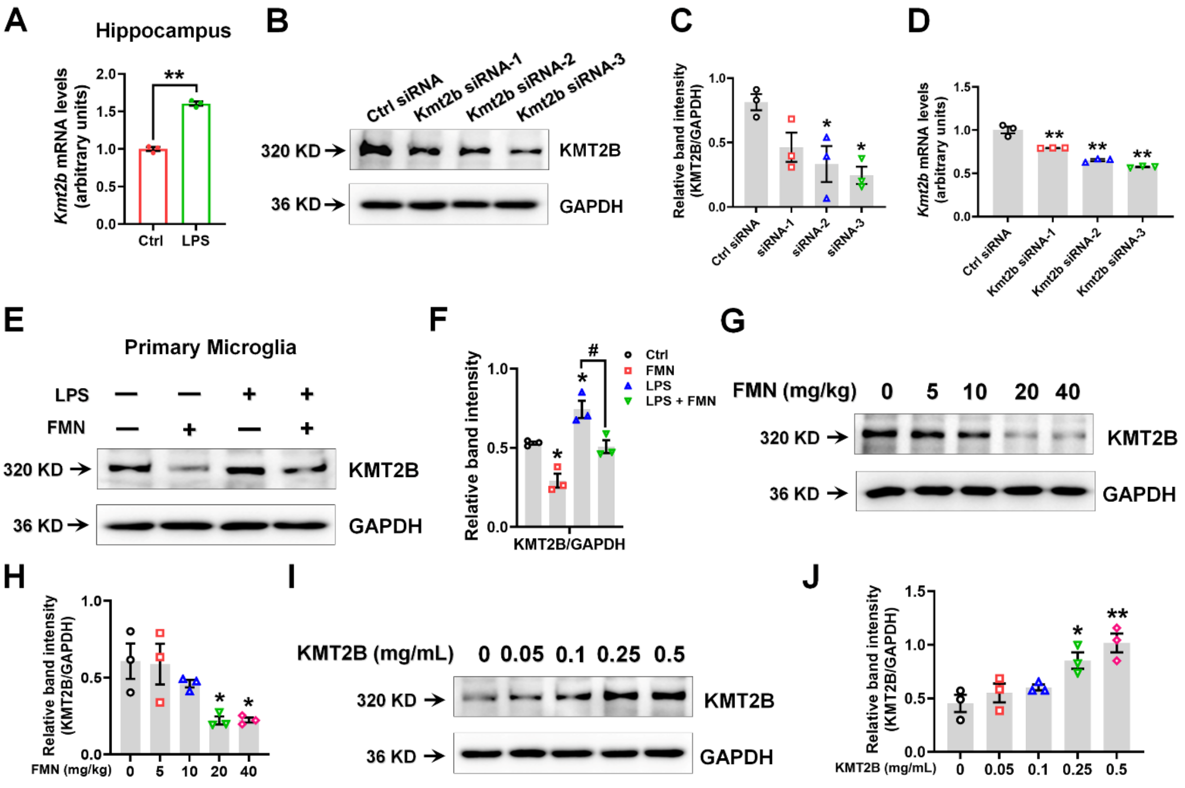

Figure S9. The effects of FMN on KMT2B expression. (A) *Kmt2b* mRNA expression levels in the hippocampus of Ctrl mice and the LPS mouse model. n = 3 per group. (B and C) Representative blots and quantification showing KMT2B expression levels in primary microglia treated with Control siRNA and three targeting RFK siRNAs. n = 3 per group. (D) *Kmt2b* mRNA expression levels in primary microglia treated with Control siRNA and three targeting RFK siRNAs. n = 3 per group. (E and F) Representative blots and quantification of KMT2B expression levels in LPS-treated primary microglia with 200 µM FMN. n = 3 per group. (G and H) Representative blots and quantification showing KMT2B expression in the hippocampus of WT mice intraperitoneally injected with different doses of FMN (0, 5, 10, 20, 40 mg/kg). n = 3 per group. (I and J) Representative blots and quantification showing KMT2B expression in microglia treated with different doses of KMT2B recombinant protein (0, 0.05, 0.1, 0.25, and 0.5 mg/mL). n = 3 per group. Results are expressed as mean ± SEM. $^{**}p < 0.01$, $^{*}p < 0.05$ vs. Control or Ctrl siRNA; $^{\#}p < 0.01$ vs. LPS. Statistical significance was determined using one-way ANOVA and Tukey's tests for *post hoc* comparisons.

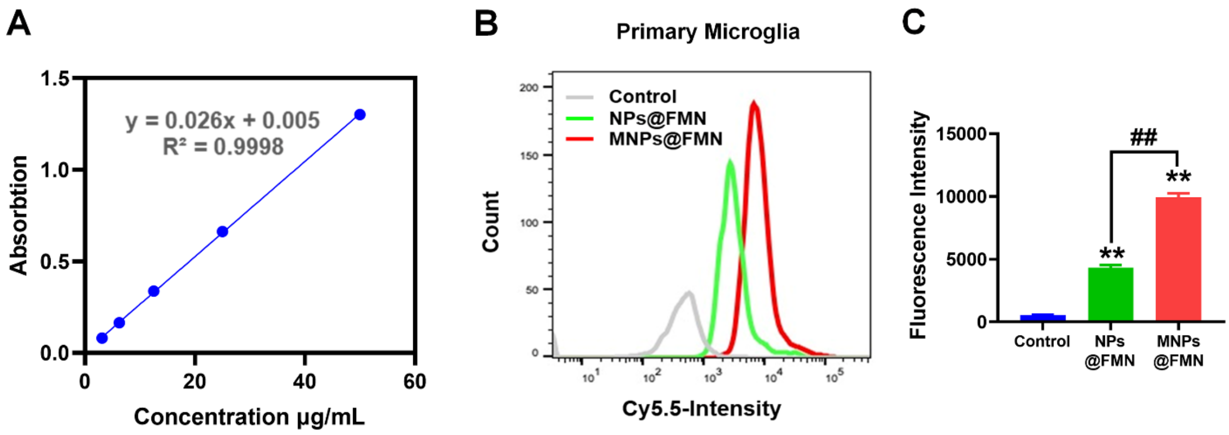

Figure S10. Characterization of MNPs@FMN. (A) UV-vis spectroscopy was used to the determine the concentration of FMN, and the standard curves were linear over the range of 3.13–50 μg/mL for FMN. (B and C) Flow cytometry analysis of cellular uptake of NPs@FMN and MNPs@FMN in primary microglia. n = 3 per group. Results are expressed as mean ± SEM. $^{**}p < 0.01$ vs. Control; $^{\#\#}p < 0.01$ vs. NPs@FMN. Statistical significance was determined using one-way ANOVA and Tukey's tests for *post hoc* comparisons.

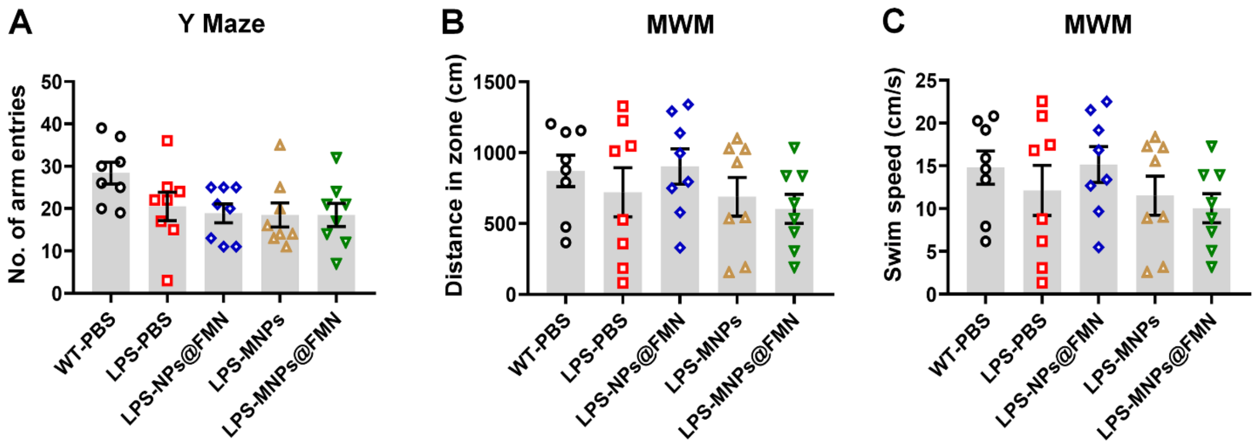

Figure S11. Behavioral tests of LPS-induced mice treated with MNPs@FMN. (A) Number of arm entries in the Y maze for LPS mice treated with NPs@FMN, MNPs or MNPs@FMN. (B and C) Distance in the pool and swim speed in the probe test for LPS mice treated with NPs@FMN, MNPs and MNPs@FMN. n = 8 per group. Results are expressed as mean ± SEM. Statistical significance was determined using one-way ANOVA and Tukey's tests for *post hoc* comparisons.

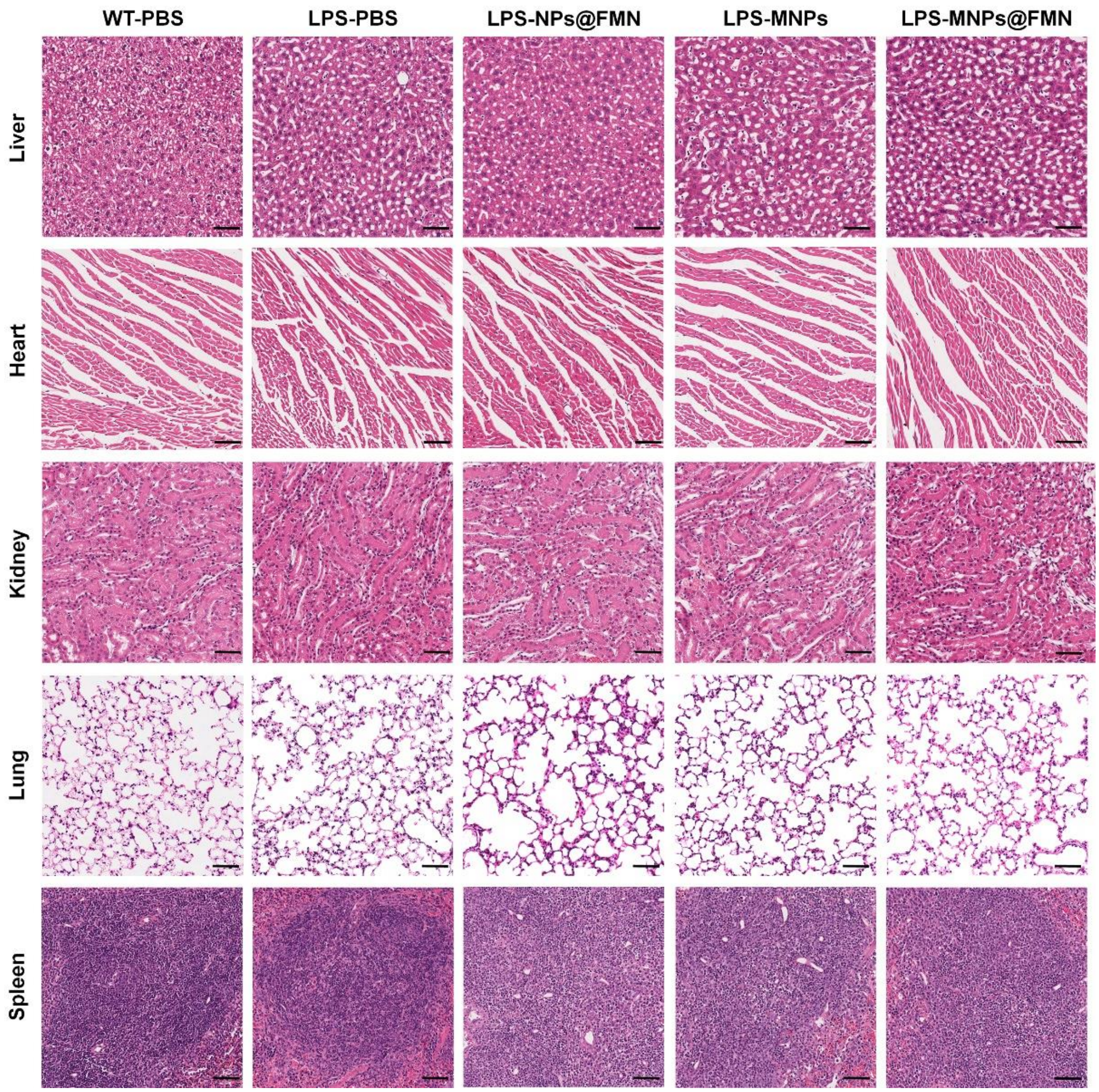

Figure S12. MNPs@FMN exerts no observable toxicity in major organs. Representative images of hematoxylin and eosin (HE) staining of major organs (including liver, heart, kidney, lung and spleen) excised from WT-PBS, LPS-PBS, LPS-NPs@FMN, LPS-MNPs and LPS-MNPs@FMN groups, n = 3 per group. There were no detectable pathological changes in the major organs derived from LPS mice treated with MNPs@FMN nanoparticles. Scale bars, 100 μm.

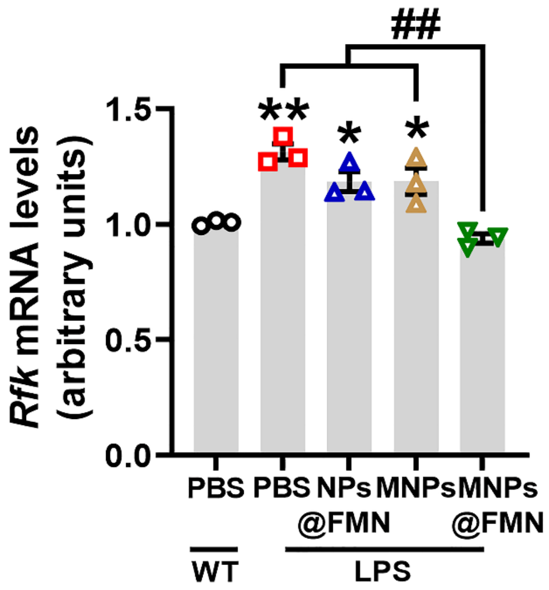

Figure S13. Effect of MNPs@FMN on *Rfk* mRNA expression in LPS-treated mice. The mRNA expression levels of *Rfk* in the hippocampus of LPS mice treated with NPs@FMN, MNPs or MNPs@FMN. n = 3 per group. Results are expressed as mean ± SEM. $^{**}p < 0.01$, $^{*}p < 0.05$ vs. WT-PBS; $^{\#\#}p < 0.01$ vs. LPS-MNPs@FMN. Statistical significance was determined using one-way ANOVA and Tukey's tests for *post hoc* comparisons.

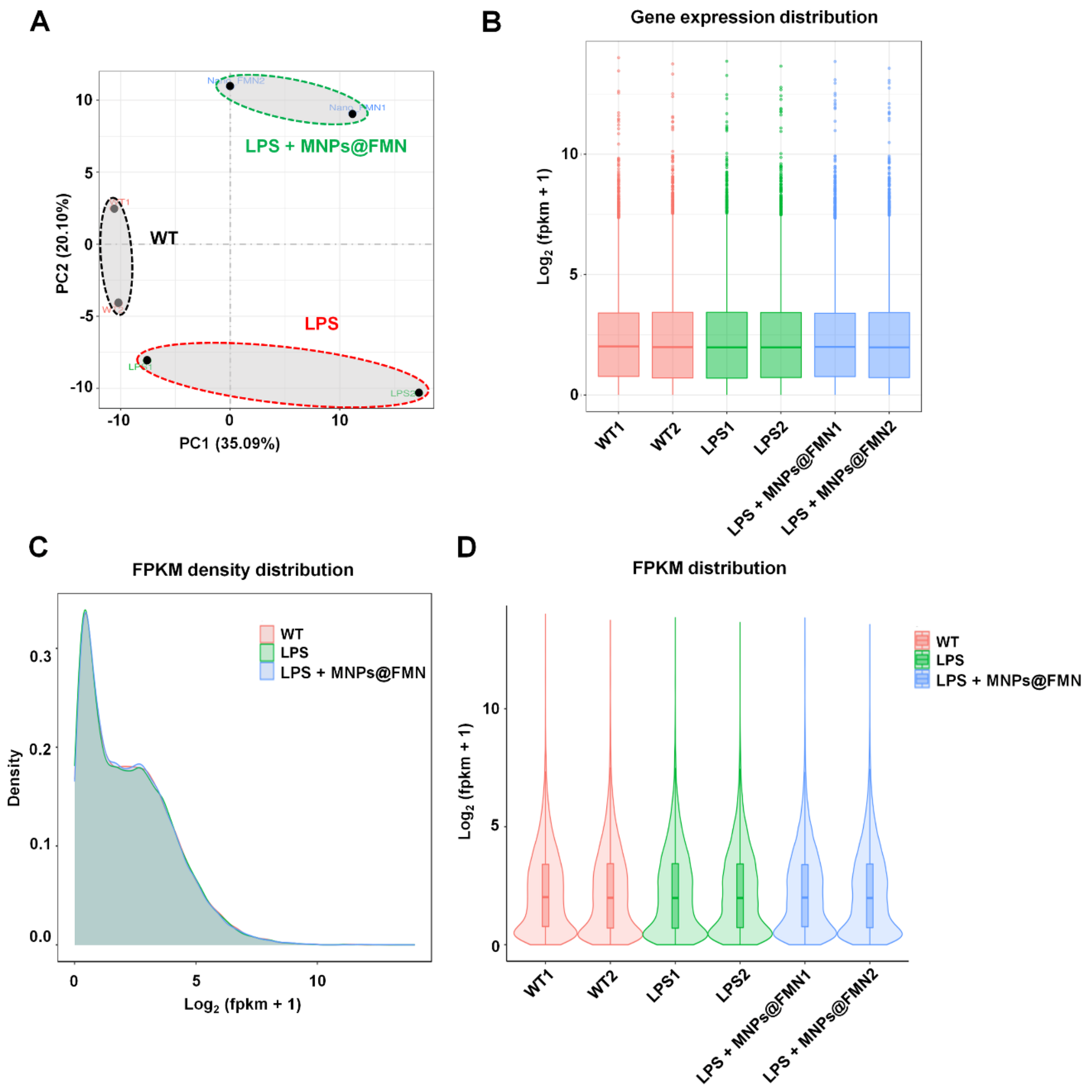

Figure S14. Gene expression distribution for genes in the RNA-seq analysis. (A) PCA score plots revealed a distinct separation of components in the WT, LPS and LPS + MNPs@FMN-treated mice. (B) Individual gene expression distribution in the WT, LPS and LPS + MNPs@FMN groups. (C and D) FPKM density distribution and individual FPKM distribution in the WT, LPS and LPS + MNPs@FMN groups.

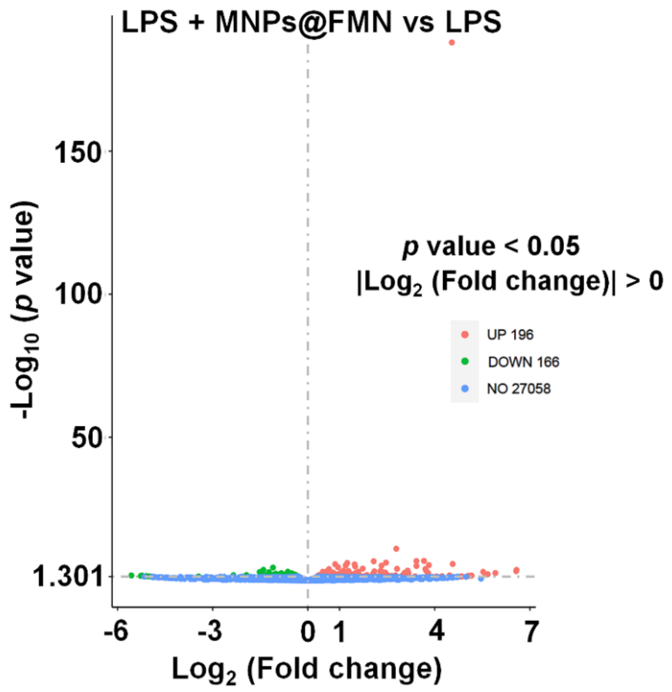

Figure S15. MNPs@FMN reverses the LPS-decreased DEGs. Volcano plot showing the DEGs between LPS + MNPs@FMN and LPS-treated mice.

Table S1. Analysis of liver and kidney function following NPs@FMN, MNPs and MNPs@FMN administration in LPS mice.

	WT-PBS (n = 4)	LPS-PBS (n = 4)	LPS-NPs@FMN (n = 4)	LPS-MNPs (n = 4)	LPS-MNPs@FMN (n = 4)	*P* Value
**AST**	86.50 (3.62)	84.50 (6.30)	106.00 (3.08)	104.50 (5.95)	107.50 (11.11)	0.058
**ALT**	32.00 (1.78)	32.50 (2.36)	44.25 (10.42)	28.00 (0.91)	30.00 (2.74)	0.225
**AST/ALT**	2.74 (0.25)	2.63 (0.22)	2.80 (0.62)	3.73 (0.15)	3.67 (0.52)	0.175
**TP**	60.20 (0.49)	58.20 (0.95)	55.48 (0.58)	54.18 (4.71)	55.60 (0.83)	0.348
**ALB**	22.20 (0.71)	21.68 (0.40)	19.93 (1.55)	19.00 (2.53)	18.28 (0.26)	0.255
**GLOB**	38.00 (0.89)	36.53 (0.59)	38.05 (0.83)	35.18 (0.92)	37.33 (0.59)	0.098
**A/G**	0.59 (0.03)	0.59 (0.01)	0.52 (0.03)	0.55 (0.09)	0.49 (0.00)	0.452
**ALP**	121.25 (1.44)	171.00 (13.24)	72.75 (5.17)	76.00 (1.15)	88.25 (13.81)	.000
**GLU**	5.44 (0.38)	5.97 (0.70)	4.51 (0.67)	5.30 (0.31)	4.93 (0.49)	0.411
**UREA**	8.43 (0.53)	7.46 (0.30)	6.80 (0.46)	7.33 (0.33)	8.02 (0.44)	0.112
**CR**	12.25 (1.03)	10.25 (0.25)	12.25 (0.48)	11.00 (0.41)	11.00 (0.41)	0.109
**UA**	378.75 (7.44)	424.75 (13.31)	374.50 (43.46)	365.50 (4.73)	391.75 (21.04)	0.423
**LDH**	704.25 (33.57)	741.75 (67.08)	554.50 (37.83)	383.25 (28.50)	671.25 (57.80)	.001

AST: aspartate aminotransferase; ALT: alanine aminotransferase; AST/ALT: aspartate aminotransferase alanine aminotransferase ratio; TP: Total protein; ALB: albumin; GLOB: globulin; A/G: albumin globulin ratio; ALP: alkaline phosphatase; GLU: blood glucose: UREA: urea nitrogen; CR: creatinine; UA: uric acid; LDH: lactate dehydrogenase. Results are expressed as the mean ± SEM. n = 4 per group. Statistical significance was determined by one-way ANOVA and Tukey tests for *post-hoc* comparisons.

Table S2. Primer used for qRT-PCR.

Mouse gene	Primer sequence (5′-3′)
Rfk	F: TGGAAACACACATCATCCATACC
	R: CACCTTGAATTGCAGAAATAAGTGAC
Il-1b	F: AATGCCACCTTTTGACAGTGAT
	R: TGCTGCGAGATTTGAAGCTG
Il-6	F: AGGATACCACTCCCAACAGACC
	R: AAGTGCATCATCGTTCATACA
Tnfa	F: CACGTCGTAGCAAACCACC
	R: TGAGATCCATGCCGTTGGC
Ifng	F: TGGCAGGAGATGTCTACACT
	R: GAAGCACCAGGTGTCAAGTC
Tgfb	F: ATTCCTGGCGTTACCTTGG
	R: AGCCCTGTATTCCGTCTCCT
Csf1r	F: CCTCAAACGTGGAGACACCAA
	R: CGTGTGCCAACATCATTGCT
Cx3cr1	F: CAACCCCTTTATCTACGCCTT
	R: GACCCATCTCCCTCGCTTG
Tmem119	F: CTGACATTCTGGCTGCTACC
	R: CACCCTTCACAGGCTTTGCTC
P2ry12	F: TTTGCTGGGCTCATCACGAAC
	R: ACTGAAGTAACTTGGCACACC
Uqcrb	F: ACTCATGAAACTGGGAGAGAAG
	R: GTCATATTTCACTTGGCCGTTT
Cox8b	F: GAAGTTCACAGTGGTTCCCAAA
	R: TTTTATAGCTCTCCAAGTGGGC
Calml4	F: ACTCATGAAACTGGGAGAGAAG
	R: GTCATATTTCACTTGGCCGTTT
Gapdh	F: ACGGGAAGCTCACTGGCATGGCCTT
	R: CATGAGGTCCACCACCCTGTTGCTG

F, forward primer sequence; R, reverse primer sequence.
